# *Polygala tenuifolia* Willd. Extract delays non-alcoholic fatty liver disease progression in rats via the COX2 and PERK-elF2α-ATF4 pathway

**DOI:** 10.3389/fphar.2025.1595752

**Published:** 2025-06-12

**Authors:** Zhiliang Sun, Zhongshi Zhou, Kezheng Liao, Yanju Liu, Yanyun Liu, Chaoyang Wang, Zhihua Zhang, Li Wen

**Affiliations:** ^1^ School of Basic Medicine, Hubei University of Chinese Medicine, Wuhan, China; ^2^ School of Pharmacy, Hubei University of Chinese Medicine, Wuhan, China; ^3^ Hubei Shizhen Laboratory, Wuhan, Hubei, China

**Keywords:** *Polygala tenuifolia*, NAFLD, PERK-ELF2α-ATF4, inflammation, Chinese medicine

## Abstract

**Background:**

Non-alcoholic fatty liver disease (NAFLD), a chronic liver disease posing a severe threat to human health, currently lacks specific therapeutic drugs. Our previous studies have demonstrated that *Polygala tenuifolia* Willd. Extract (EPT) can significantly suppress the PGE2 inflammatory pathway, thereby exerting anti-inflammatory and antioxidant effects, as well as improving lipid metabolism. These findings suggest that EPT might hold potential value for the prevention and treatment of NAFLD.

**Purpose:**

This study aims to investigate the role of EPT in NAFLD and its multi - target synergistic mechanisms, and to preliminarily explore its impact on the early progression of NAFLD.

**Methods:**

The detection of EPT components in rat blood was performed by UPLC-MS/MS. A rat NAFLD model was established using a high-fat emulsion gavage method, and pathological changes in liver tissue were assessed via H&E, Oil Red O, and Sirius Red staining. RNA-seq, immunohistochemistry, RT-qPCR, and Western blotting were used to evaluate the expression of cyclooxygenase 1 (COX1), cyclooxygenase 2 (COX2), inflammatory factors (IL-1, IL-6, TNF-α), oxidative stress-related proteins in the KEAP1 pathway, proteins involved in the protein kinase R-like endoplasmic reticulum kinase (PERK) pathway, and apoptosis-related proteins (Bcl-2 and Caspase-3).

**Results:**

EPT significantly attenuated the histopathological alterations in liver tissue and ameliorated lipid accumulation in the livers of NAFLD rats. RNA-seq identified differential changes in the arachidonic acid metabolism, inflammatory, and endoplasmic reticulum-related pathways. Immunohistochemistry showed that EPT reversed the elevated expression of COX2, the ER stress marker GRP78, and PERK in the liver tissue of model rats. RT-qPCR and Western blot analyses confirmed that EPT reduced the expression of COX1, COX2, IL-1β, IL-6, TNF-α, and KEAP1, while increasing Nrf2 and HO-1 levels. Furthermore, EPT downregulated GRP78, PERK, p-PERK, eIF2α, p-eIF2α, ATF4, CHOP, Bcl-2, and Caspase-3.

**Conclusion:**

These results suggest that EPT might exert anti - inflammatory and antioxidant effects by modulating the COX2 pathway in NAFLD rats, downregulate the ER stress PERK - eIF2α - ATF4 signaling pathway, alleviate ER stress, inhibit apoptosis, and delay NAFLD progression.

## 1 Introduction

Non-alcoholic fatty liver disease (NAFLD) is one of the most prevalent chronic liver diseases, characterized by abnormal lipid accumulation in the liver, accompanied by inflammation and oxidative stress (Sahu et al., 2023). Its incidence is on the rise globally, posing a significant threat to human health (Riazi et al., 2022). Current treatment strategies primarily focus on lifestyle modifications and managing metabolic disorders such as obesity and diabetes. Pharmacological interventions, including pioglitazone, metformin, and simvastatin, have demonstrated efficacy in addressing insulin resistance, regulating lipid metabolism, and reducing liver fat accumulation. However, these treatments have limited success in mitigating inflammation and oxidative stress. Recent studies have highlighted the central roles that these two processes play in the process of NAFLD progression, underscoring the need for new therapeutic interventions.

Fat accumulation in the liver triggers local inflammatory responses, activating macrophages and inducing the release of pro-inflammatory cytokines such as IL-1β, IL-6, and TNF-α. These cytokines not only damage hepatocytes but also promote reactive oxygen species (ROS) production, leading to oxidative stress. Excessive ROS, in turn, activate pathways such as NF-κB, further amplifying inflammation (Das et al., 2024; Taru et al., 2024). In addition, inflammation and oxidative stress can exacerbate lipotoxicity, trigger ER stress (ERS), promote apoptosis, and further drive the progression of NAFLD to non-alcoholic steatohepatitis (NASH) (Arroyave-Ospina et al., 2021). This vicious cycle accelerates the progression of NAFLD from simple steatosis to non-alcoholic steatohepatitis and liver fibrosis, potentially culminating in cirrhosis or hepatocellular carcinoma. Thus, in - depth research on the roles of inflammation and oxidative stress in NAFLD development and the relevant prevention and treatment strategies, as well as exploration of ER stress’ role in NAFLD, is crucial for delaying NAFLD progression.

*Polygala tenuifolia* Willd. (PTW) is a traditional Chinese medicine known for its lipid-lowering, anti-inflammatory, and antioxidant properties. Our previous research has shown that the active component of PTW extract (EPT), specifically the total saponins derived from *Polygala*, exhibits significant lipid-lowering effects and mitigates lipid accumulation in rats (Wen et al., 2015). Our previous study showed that EPT can exert anti - inflammatory and antioxidant effects by regulating the TLR4/NF-κB signaling pathway, reducing neutrophil counts and pro - inflammatory factors like IL - 1β, thus alleviating acute lung injury in rats (Guo et al., 2024). We also confirmed that EPT significantly inhibits PGE2, a downstream product of COX2. Given the close relationship between lipid metabolic disorder, inflammation, oxidative stress and ERS in driving NAFLD progression, investigating EPT’s regulatory effects on COX2 and ERS might offer new therapeutic insights. This experiment aims to explore EPT’s dual effects on COX2 and ERS and whether they can delay NAFLD progression.

In this study, we established an NAFLD rat model using high-fat emulsion gavage. We employed network pharmacology and transcriptomics to screen the targets and pathways associated with EPT, to preliminarily explore the molecular mechanisms of early NAFLD development.

## 2 Materials and methods

### 2.1 Materials and reagents

Methanol and acetonitrile used for UPLC-MS/MS were purchased from Merck (Darmstadt, Germany), while formic acid was purchased from Aladdin (Seattle, WA, United States. Cholesterol, lard, sodium cholate, Tween 80, 1,2-propanediol, and propylthiouracil were provided by Shanghai McLean Biochemical Technology Co., Ltd. (Shanghai, China). Total cholesterol (TC) and triglyceride (TG) assay kits were provided by Jiancheng Bioengineering Institute (Nanjing, China). The aspartate aminotransferase (AST), alanine aminotransferase (ALT), malondialdehyde (MDA), and superoxide dismutase (SOD) assay kits were provided by Eliyure Bio-Tech Co., Ltd. (Wuhan, China).

### 2.2 The preparation of EPT

PTW was sourced from Hubei Pharmaceutical Company (China; batch no. 20141120), PTW was verified by Dr. Hongbin Yang from the Hubei University of Chinese Medicine. The material was decocted three times, and the resulting solution was concentrated to 1 g/mL. The concentrate was subjected to D101 macroporous adsorption resin column chromatography, sequentially eluted with purified water, 30% ethanol, and 70% ethanol, and the 70% ethanol eluate was vacuum-dried to obtain EPT powder. Based on a raw material-to-EPT ratio of 3.13 g:100 mg, the appropriate concentration of aqueous solution was prepared for analysis.

### 2.3 Animals

Male Sprague-Dawley (SD) rats (180–200 g) were obtained from the Hubei Provincial Experimental Animal Research Center (Wuhan, China; Animal Certificate No. 42000600050367). Rats were housed under specific pathogen-free (SPF) conditions at the Hubei University of Chinese Medicine, with controlled temperature (23°C ± 2°C), humidity (65% ± 5%), and a 12-h light/dark cycle (lights on from 08:00 to 20:00). Animals had free access to food and water. All experimental procedures complied with Chinese and international guidelines and were approved by the Animal Ethics Committee of Hubei University of Chinese Medicine (Ethics Approval No. HUCMS00301638).

### 2.4 UPLC-MS/MS analyses of serum EPT components

After 1 week of acclimation, six rats were randomly divided into two groups: a blank group and an EPT treatment group. The blank group received daily oral gavage with PBS, while the EPT treatment group received EPT (100 mg/kg) by oral gavage. After 7 days, the rats were anesthetized 1 hour post-administration, and blood samples were collected from the abdominal aorta. The blood samples were allowed to stand at 4°C for 12 h, followed by centrifugation (4°C, 3,000 rpm, 10 min). The serum was then transferred to fresh centrifuge tubes and stored at −80°C until further analysis.

For UPLC-MS/MS analysis, the stored serum was thawed on ice and vortexed for 10 s. A 50 μL serum sample was mixed with 300 μL of 20% acetonitrile-methanol extraction solution containing an internal standard. The mixture was centrifuged (4°C, 12,000 rpm, 3 min), and 200 μL of the supernatant was transferred to a fresh tube. After standing at −20°C for 30 min, the sample underwent a second centrifugation (4°C, 12,000 rpm, 3 min), and 180 μL of the supernatant was transferred to an injection vial for LC-MS/MS analysis.

The chromatographic column used for UPLC analysis was an Agilent SB-C18 (1.8 μm, 2.1 mm × 100 mm). The flow rate was set at 0.35 mL/min, the column temperature at 40°C, and the injection volume at 2 μL. The mobile phase consisted of (A) 0.1% formic acid aqueous solution and (B) 0.1% formic acid acetonitrile solution. The gradient program was as follows: 0–9 min, 95% A/5% B; 9–10 min, 5% A/95% B; 11–14 min, 95% A/5% B. Wastewater was directed alternately to an ESI-QTRAP for analysis. The ESI source parameters were as follows: source temperature, 500°C; IS, 5500 V (positive mode) or −4500 V (negative mode); GSI, GSII, and CUR were 50, 60, and 25 psi, respectively; CAD was set to high, and the collision gas to medium.

### 2.5 Network pharmacology

Active compound targets were predicted using TCMSP (https://old.tcmsp-e.com/tcmsp.php), DrugDesign (http://www.swisstargetprediction.ch/), and SymMap (https://www.symmap.org/). Disease-related targets associated with EPT were identified using DisGeNET (https://www.disgenet.org/). An intersection analysis was conducted between compound and disease targets to identify shared targets, which were subjected to KEGG pathway enrichment analysis. Cytoscape 3.7.0 was used to construct a compound-target-pathway network diagram.

### 2.6 Animal model assays

Following a 1-week acclimation period, 50 rats were randomly assigned to five groups (10 rats each): normal group, high-fat model group, EPT low-dose group (EPT-L, 50 mg/kg), EPT high-dose group (EPT-H, 100 mg/kg), and Celecoxib group (20 mg/kg). With the exception of the normal group, all rats were gavaged daily with a high-fat emulsion (10% cholesterol, 30% lard, 2% sodium cholate, 10% Tween 80, 10% 1,2-propanediol, and 0.5% propylthiouracil) at a dose of 10 mL/kg for 4 weeks to induce NAFLD. After the modeling period, the EPT-L, EPT-H, and Celecoxib groups received their respective treatments (10 mL/kg daily) for 2 weeks, while the normal and high-fat model groups were gavaged with an equivalent volume of normal saline. At the end of the treatment period, the rats were fasted for 12 h (water excluded), anesthetized with 10% sodium pentobarbital, and blood samples were collected from the abdominal aorta using heparinized anticoagulant tubes.

### 2.7 Serum biochemistry

Blood samples were allowed to stand at 4°C for 4 h and then centrifuged (4°C, 12,000 rpm, 10 min). The supernatant was transferred to centrifuge tubes and stored on ice for analysis. Serum levels of TC, TG, AST, and ALT were measured using assay kits according to the manufacturer’s instructions.

### 2.8 Liver sample preparation

Liver tissues were excised, weighed, and photographed. A portion of each liver was fixed in 4% paraformaldehyde for histological analysis. Fixed tissues were embedded in paraffin or OCT compound, sectioned, and subjected to H&E, Oil Red O, and Sirius Red staining. Stained sections were examined microscopically, and images were captured for analysis.

### 2.9 Liver biochemistry

Liver tissues were homogenized, and the homogenates were centrifuged (4°C, 6,000 rpm, 10 min). The supernatant was transferred to centrifuge tubes and stored on ice. The levels of MDA and SOD were measured using specific assay kits as per the manufacturer’s instructions.

### 2.10 Immunohistochemical (IHC) staining

Paraffin-embedded liver sections were deparaffinized and rehydrated. Sections were incubated with primary antibodies specific for COX2 (Affinity, AF7003), GRP78 (Affinity, AF5366), and PERK (Affinity, AF5304) at 37°C for 1 h. An HRP-conjugated goat anti-rabbit IgG secondary antibody (Abcam, ab6721) was then applied, and sections were incubated at room temperature in the dark for 30 min. Following washes with PBS (pH 7.4), sections were treated with 5 μg/mL DAB at room temperature for 30 min. Slides were mounted and examined under a confocal microscope, capturing appropriate images.

### 2.11 RNA-seq

Total RNA was extracted from liver tissues using the TRIzol method. RNA quality and concentration were assessed using a Qubit fluorometer and a Qsep400 high-throughput biofragment analyzer. After fragmenting RNA with an appropriate fragmentation buffer, it was reverse-transcribed into cDNA, purified with magnetic DNA-binding beads, and ligated with sequencing adapters. Adapter-ligated libraries were amplified via RT-qPCR and sequenced using an Illumina instrument.

### 2.12 Western blotting (WB)

Liver tissues were homogenized, and total protein was extracted using RIPA lysis buffer with 2% phosphatase inhibitor. Proteins were separated via SDS-PAGE, transferred to PVDF membranes, and blocked with 5% nonfat milk for 1 h. Membranes were then incubated overnight at 4°C with primary antibodies (Table 1). After washing with TBST (3 washes, 5 min/wash), HRP-conjugated secondary anti-IgG (Abcam, ab6721) was used to probe these blots for 30 min. After washing again with TBST as above, an enhanced chemiluminescence reagent (Biosharp, China) was used for detection, developing the blots in a darkroom. The images were scanned and analyzed.

**TABLE 1:** The information of antibodies used in the present experiment.

Antibody	Species	Dilutions WB IF	Company
GRP78	Rabbit	1:10000	Affinity, United States (AF5366)
PERK	Rabbit	1:10000	Affinity, United States (AF5304)
p-PERK	Rabbit	1:10000	Affinity, United States (DF7576)
elF2α	Rabbit	1:10000	Affinity, United States (AF6087)
p-elF2α	Rabbit	1:10000	Affinity, United States (AF3087)
ATF4	Rabbit	1:10000	Affinity, United States (DF6008)
CHOP	Rabbit	1:10000	Affinity, United States (AF5280)
COX1	Rabbit	1:10000	Affinity, United States (AF7002)
COX2	Rabbit	1:10000	Affinity, United States (AF7003)
beta-Actin	Rabbit	1:10000	Affinity, United States (T0022)
Keap1	Rabbit	1:1000	Proteintech, United States (10503-2-AP)
Nrf2	Rabbit	1:1000	Proteintech, United States (16396-1-AP)
HO-1	Rabbit	1:1000	Proteintech, United States (10701-1-AP)
IL-1β	Rabbit	1:10000	Zenbio, China (516288)
IL-6	Rabbit	1:10000	Zenbio, China (500286)
TNF-α	Rabbit	1:1000	Zenbio, China (346654)
Bcl-2	Rabbit	1:1000	Zenbio, China (381702)
Caspase-3	Rabbit	1:1000	Zenbio, China (341034)

### 2.13 Real-time quantitative PCR(RT-qPCR)

Total RNA was extracted using TRIzol reagent (Vazyme, China). RNA was reverse-transcribed using a reverse transcription kit (ABclonal, China) with the following conditions: 50°C for 15 min and 85°C for 5 min. For RT-qPCR, amplification was performed using SYBR Green (Vazyme, China) under the following conditions: 95°C for 30 min, 95°C for 10 min, and 60°C for 30 min, with 39 cycles. GAPDH served as the internal control. Data were analyzed using the 2^−ΔΔCt^ method, with primer sequences listed in Table 2.

**TABLE 2:** Primers used for RT-qPCR.

Gene	Forward (5′−3′)	Reversed (5′−3′)
GRP78	TGA​AGT​TCA​CTG​TGG​TGG​CG	CCA​AGT​CAA​TGC​CAA​CCA​CC
PERK	TGT​CTT​GGT​TGG​GTC​TGA​TG	CCT​TCT​TGC​GGA​TGT​TCT​TG
elf2α	CGA​GGA​CAA​ATG​GAA​GTA​TG	GGC​TGT​TAA​GAT​ATG​CTC​AC
ATF4	AAT​GGA​TGA​CCT​GGA​AAC​C	GGG​CTC​CTT​ATT​AGT​CTC​TTG
CHOP	TCG​CCT​TTG​AGA​CAG​TGT​CC	TGT​GGT​CTC​TAC​CTC​CCT​GG
Bcl-2	GAG​GGG​CTA​CGA​GTG​GGA​TA	TCA​AAC​AGA​GGT​CGC​ATG​CT
Caspase-3	GGCATCTCCTGTGATTGG	CTCAGCACTCTGGGAAAG
Keap1	CAC​TTC​GGG​GAG​GAG​GAG​TT	GGG​CAG​TCG​TAT​TTG​ACC​CA
Nrf2	GTC​AGC​TAC​TCC​CAG​GTT​GC	CAG​GGC​AAG​CGA​CTG​AAA​TG
HO-1	TTT​TCA​CCT​TCC​CGA​GCA​T	GCC​TCT​TCT​GTC​ACC​CTG​T
IL-1	TTG​AGT​CTG​CAC​AGT​TCC​CC	TCC​TGG​GGA​AGG​CAT​TAG​GA
IL-6	AGA​GAC​TTC​CAG​CCA​GTT​GC	AGT​CTC​CTC​TCC​GGA​CTT​GT
TNF-α	GGC​TTT​CGG​AAC​TCA​CTG​GA	GGG​AAC​AGT​CTG​GGA​AGC​TC
GAPDH	GTC​GGT​GTG​AAC​GGA​TTT​G	TCC​CAT​TCT​CAG​CCT​TGA​C

### 2.14 Statistical analysis

Statistical analysis was conducted using GraphPad Prism 10.0.2. One-way ANOVAs followed by Dunnett’s test were used for multiple group comparisons. A P-value <0.05 was considered statistically significant.

## 3 Results

### 3.1 Characterization of the EPT-derived compounds in the serum

**FIGURE 1:**
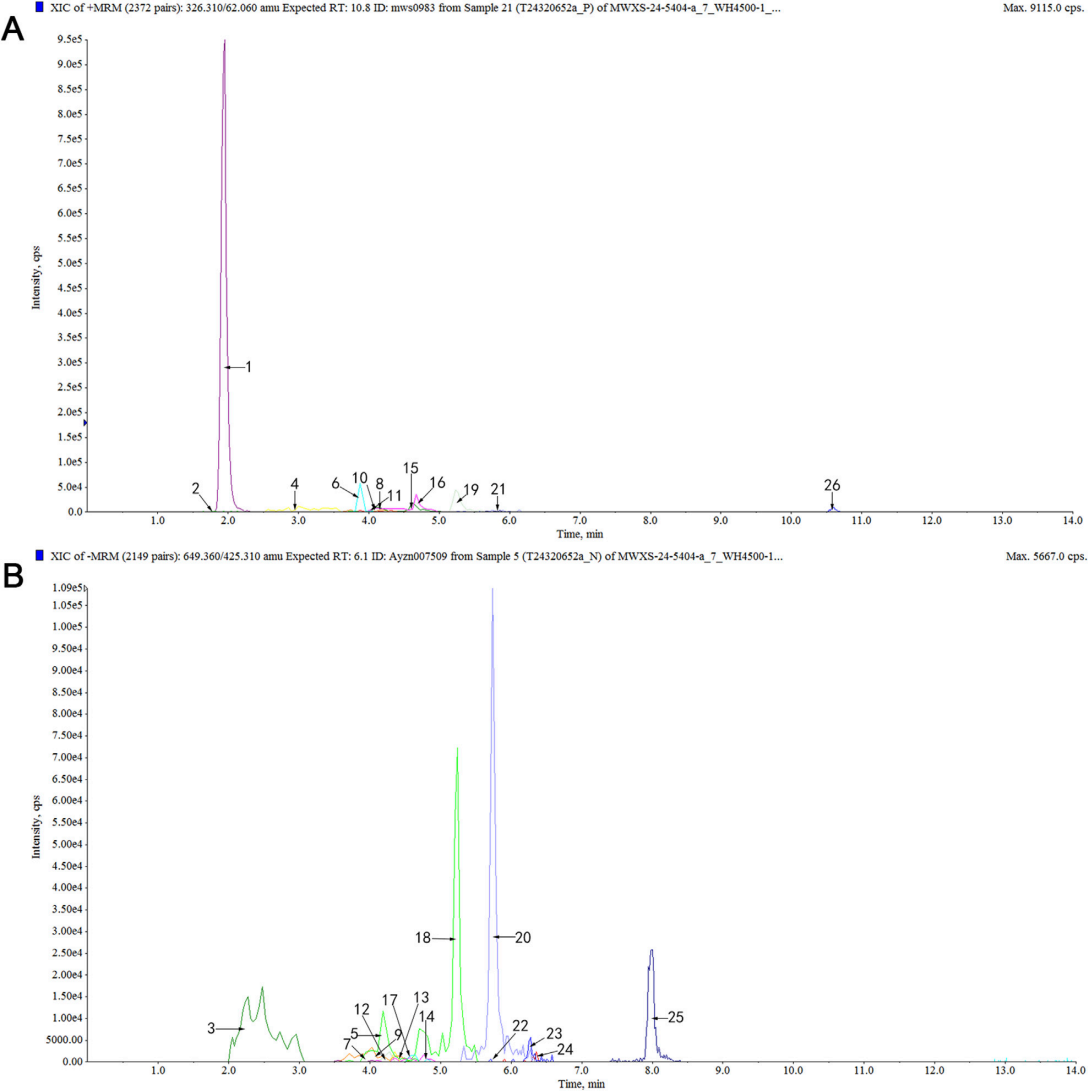
Analysis of serum differential components in rats after EPT treatment using the UPLC-MS/MS method. **(A)** XIC chromatogram in positive ion mode. **(B)** XIC chromatogram in negative ion mode. Table 3 for specific compounds.

Using a UPLC-ESI-MS/MS system, a total of 456 differential serum components were identified in rat serum after EPT administration when analyzed in both positive and negative ionization modes (Supplementary Figures S1, S2; Supplementary Table S1). These components included 10 saponins, 51 flavonoids, 56 terpenoids, 44 glycosides, 78 phenolic acids, 11 lignans, 15 coumarins, 9 quinones, 127 alkaloids, and 55 other compounds. Representative active compounds are displayed in the XIC chromatograms (Figures 1A,B), with molecular formulas and retention times detailed in Table 3.

**TABLE 3:** The main bioactive components of EPT in serum.

NO	Retention (min)	Formula	Identification
1	1.9	C_8_H_9_N	N-Benzylmethylene isomethylamine
2	2.1	C_14_H_20_O_9_	Leonuriside A
3	2.5	C_7_H_6_O_4_	3,4-Dihydroxybenzoate
4	3	C_10_H_8_O_3_	6-Methoxycoumarin
5	3.5	C_25_H_28_O_15_	Polygalaxanthone XI
6	3.9	C_25_H_28_O_14_	Polygalaxanthone IX
7	3.9	C_30_H_36_O_17_	Tenuifoliside B
8	4	C_8_H_8_O_3_	4-Hydroxy-3-methoxy-benzaldehyde
9	4.1	C_9_H_12_O_4_	Antiarol
10	4.1	C_26_H_30_O_15_	Polygalaxanthone V
11	4.2	C_11_H_10_O_5_	Isofraxidin
12	4.2	C_27_H_32_O_16_	Polygalaxanthone VII
13	4.3	C_33_H_40_O_18_	Arillanin A
14	4.4	C_10_H_12_O_5_	Eudesmic acid
15	4.4	C_11_H_12_O_4_	Sinapoyl aldehyde
16	4.5	C_21_H_18_O_11_	Baicalin
17	4.6	C_31_H_38_O_17_	Tenuifoliside A
18	5	C_12_H_14_O_5_	3,4,5-Trimethoxycinnamic acid
19	5.1	C_14_H_31_NO_2_	Tetradecasphinganine
20	5.7	C_20_H_22_O_6_	Matairesinol
21	5.8	C_16_H_12_O_7_	Capillarisin
22	5.8	C_36_H_56_O_12_	Tenuifolin
23	6.1	C_35_H_54_O_11_	Fallaxsaponin A
24	6.2	C_30_H_46_O_7_	Presenegenin
25	7.9	C_15_H_24_O_2_	2,6-Di-tert-butyl-4-hydroxymethylphenol
26	10.8	C_20_H_39_NO_2_	N-Oleoylethanolamine

### 3.2 Multivariate analysis and network pharmacology prediction of EPT

**FIGURE 2:**
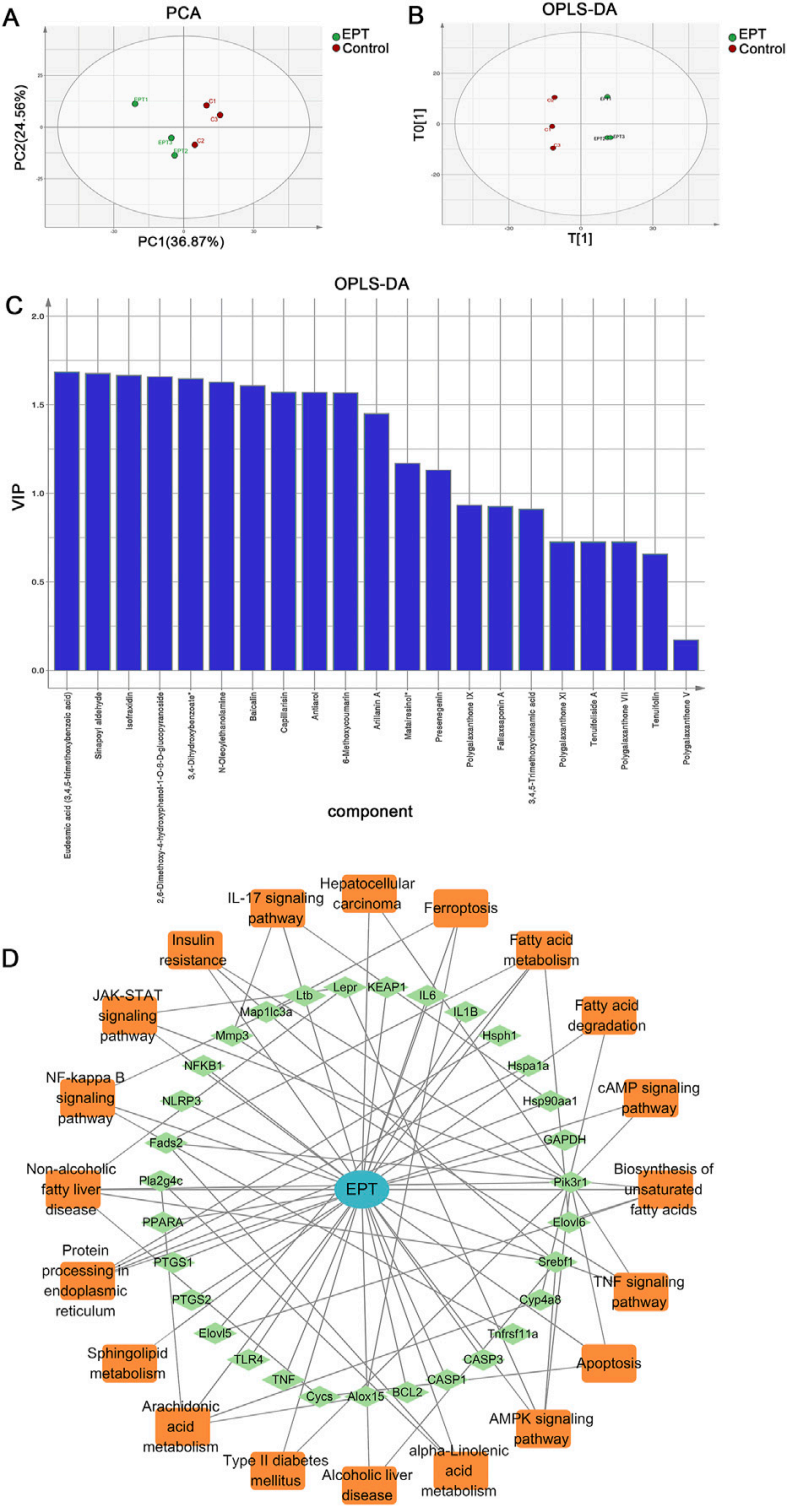
Multivariate analysis and network pharmacology prediction of EPT. **(A)** PCA analysis. **(B)** OPLS-DA analysis. **(C)** VIP ranking. **(D)** Construction of the Component-Target-Pathway network of EPT.

Principal component analysis (PCA) and orthogonal partial least squares-discriminant analysis (OPLS-DA) revealed clear distinctions between the control and EPT-treated groups, with differential metabolites ranked by VIP scores (Figures 2A–C). Network pharmacology analysis was used to construct the EPT component-target-pathway network (Figure 2D). The analysis predicted significant involvement of pathways including arachidonic acid metabolism, the NAFLD pathway, inflammation-related pathways, and endoplasmic reticulum (ER)-associated pathways.

### 3.3 EPT alleviates hepatic steatosis and liver injury in NAFLD

**FIGURE 3:**
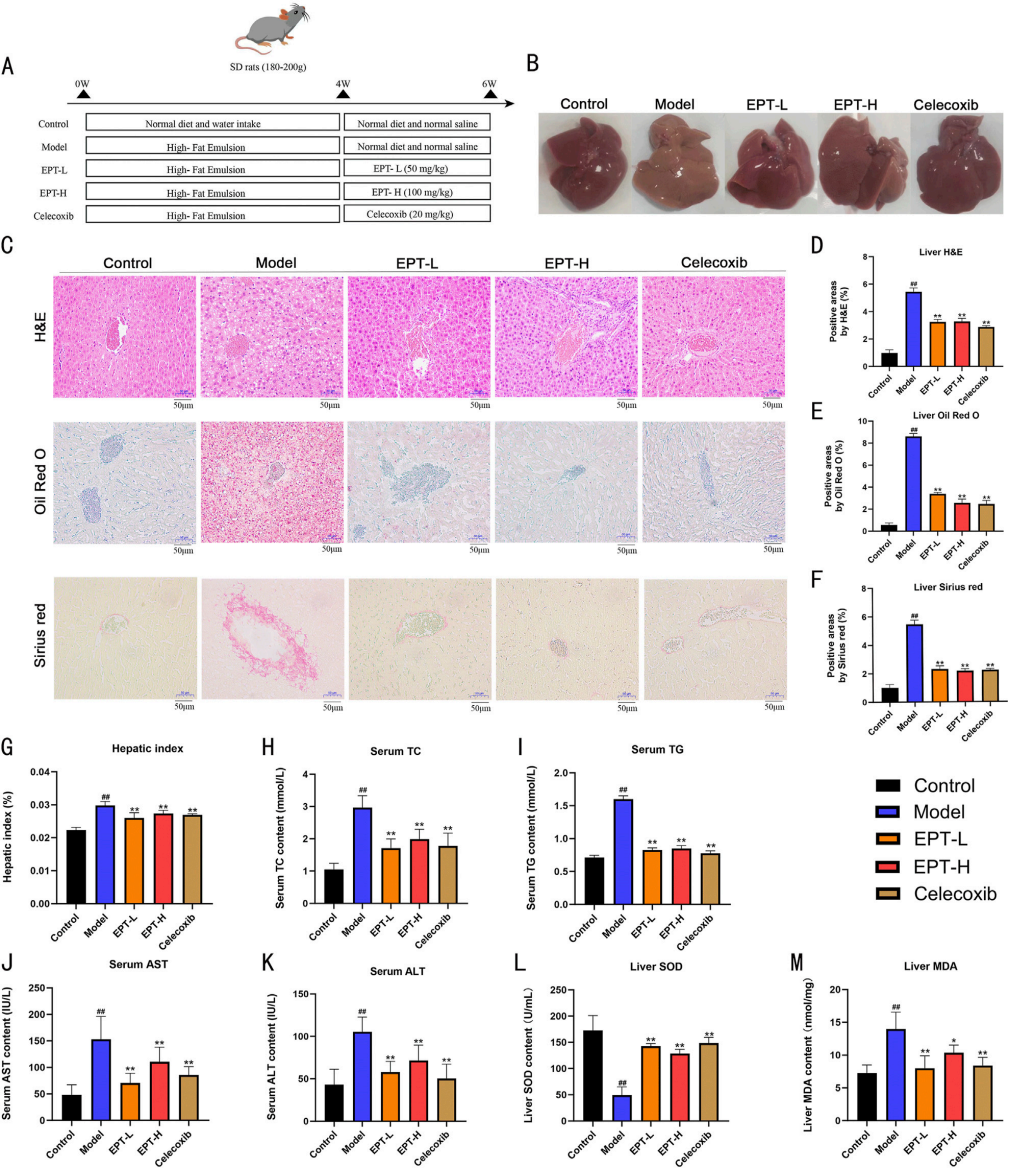
Histopathological improvement of NAFLD rats by EPT. **(A)** Flowchart of the animal experiment in this study. **(B)** Representative images of rat liver samples. **(C)** Representative images of H&E, Oil Red O, and Sirius Red staining (all images were captured at a magnification of ×200, bar = 50 μm). **(D)** Lipid vacuole quantification in H&E staining. **(E)** Quantification of lipid droplets in Oil Red O staining. **(F)** Collagen quantification in Sirius Red staining. **(G)** Liver-to-body weight ratio (%) of rats. **(H–K)** Assessment of TC, TG, AST, and ALT levels in rat serum via ELISA kits. **(L,M)** Detection of SOD and MDA levels in rat liver tissue by assay kits. Data were expressed as mean ± SD (All measurements in animal tissues were obtained from the optimal samples selected after three independent experiments, with n = 3). ^*##*^ *P* < 0.01 compared with the control group. ^*^ *P* < 0.01 compared with the model group. ^**^ *P* < 0.01 compared with the model group.

In the process of this animal experiment (Figure 3A), in the model group, rat livers exhibited yellow discoloration and significant fat accumulation compared to the control group (Figure 3B). Histological analysis using H&E staining revealed severe hepatic steatosis characterized by numerous lipid vacuoles. Oil Red O staining showed widespread lipid droplet accumulation, while Sirius Red staining demonstrated substantial collagen deposition in the liver tissue (Figures 3C–F), with a marked increase in the liver-to-body weight ratio (Figure 3G). Serum levels of TC and TG were significantly elevated in the model group (Figures 3H,I). EPT treatment notably reversed these changes, reducing serum lipid levels and alleviating hepatic steatosis. Additionally, EPT significantly decreased AST and ALT levels (Figures 3J,K), suggesting improved liver function and reduced liver injury. EPT significantly elevated hepatic SOD levels and reduced MDA levels in NAFLD rats (Figures 3L,M), this indicates that EPT has the potential to enhance antioxidant defenses and reduce oxidative stress in the liver tissue of NAFLD rats.

### 3.4 RNA-seq analysis

**FIGURE 4:**
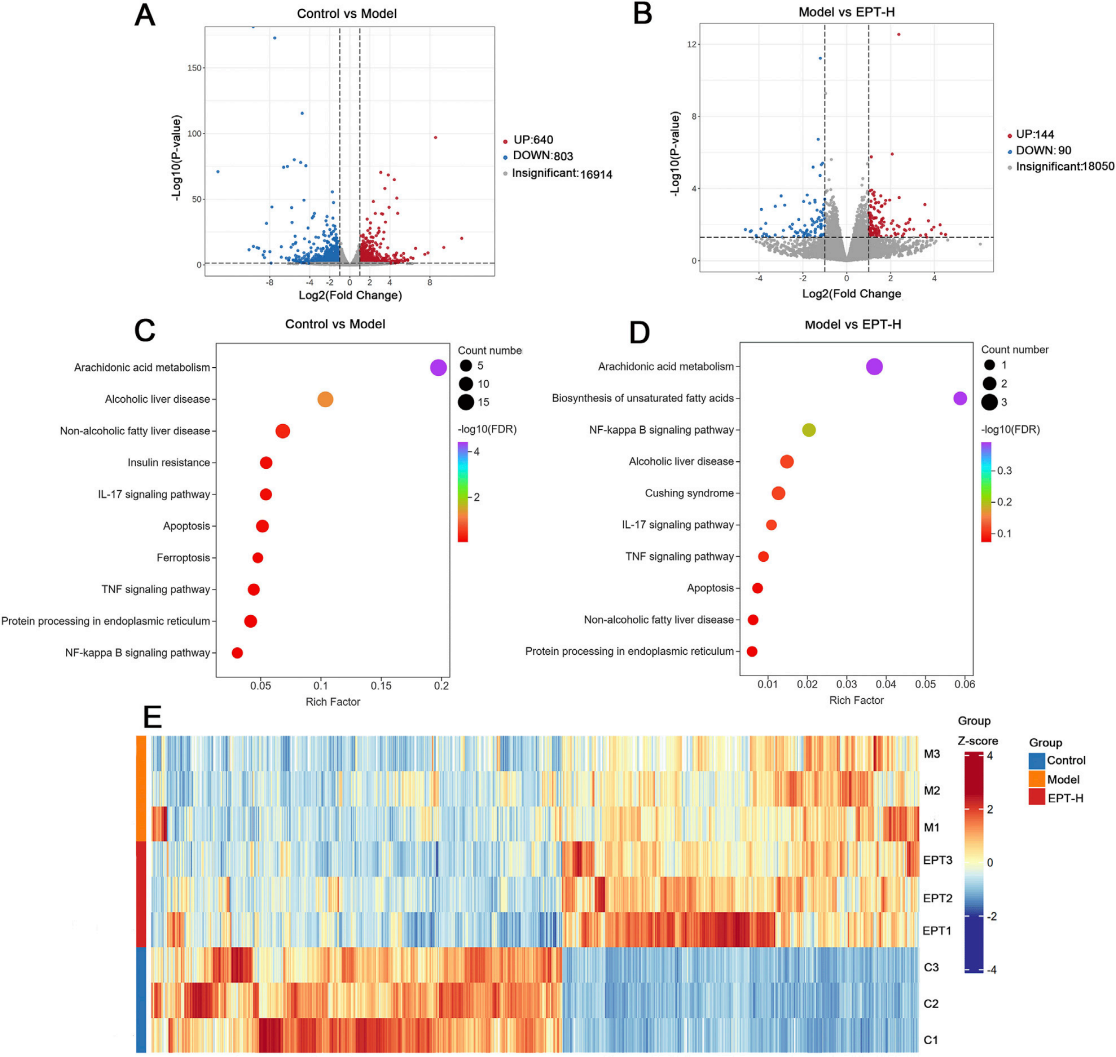
Transcriptomic analysis. **(A)** Volcano plot comparing the control group and the model group. **(B)** Volcano plot comparing the model group and the EPT group. **(C)** Bubble chart illustrating the control group *versus* the model group. **(D)** Bubble chart illustrating the model group *versus* the EPT group. **(E)** Cluster heatmap of the control group, model group, and EPT group.

To elucidate the pathways through which EPT alleviates NAFLD progression, transcriptomic analysis was conducted. Compared to the control group, the model group exhibited 640 upregulated and 803 downregulated genes. EPT treatment led to the upregulation of 144 genes and the downregulation of 90 genes. Pathways related to arachidonic acid metabolism, NAFLD, inflammation, and ER stress were identified as significantly altered (Figures 4A–E).

### 3.5 EPT modulates COX2, GRP78, and PERK expression in the liver of NAFLD model rats

**FIGURE 5:**
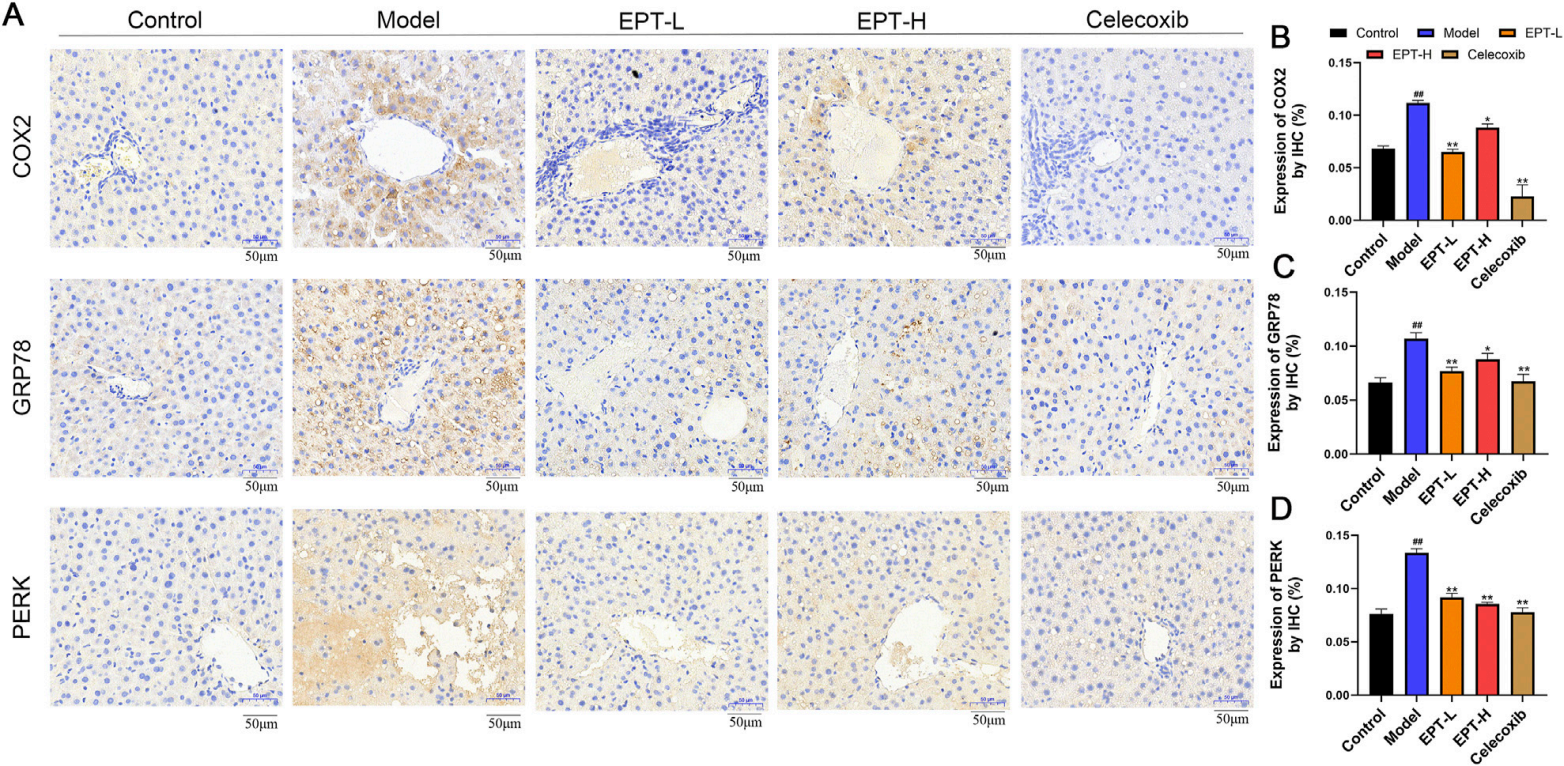
Detection of COX2, GRP78, and PERK expression in rat liver tissue by immunohistochemical staining. **(A)** Results of immunohistochemical staining. **(B–D)** Quantitative expression of COX2, GRP78, and PERK in rat liver tissue by immunohistochemistry. ^##^ *P* < 0.01 compared with the control group. ^*^ *P* < 0.05, ^**^ *P* < 0.01 compared with the model group.

To clarify the impact of EPT on the pathways found to be differentially enriched in the above analyses, IHC staining was conducted. These analyses revealed significantly increased expression of COX2 and ER stress markers GRP78 and PERK in the liver tissue of the model group (Figures 5A–D). These findings suggest that there might be a potential interaction between COX2 and ER stress via the PERK pathway in the NAFLD model. Following EPT treatment, the expression of COX2, GRP78, and PERK was significantly reduced, indicating that EPT alleviates ER stress by downregulating COX2 expression.

### 3.6 EPT mitigates inflammation and oxidative stress in NAFLD model rats

**FIGURE 6:**
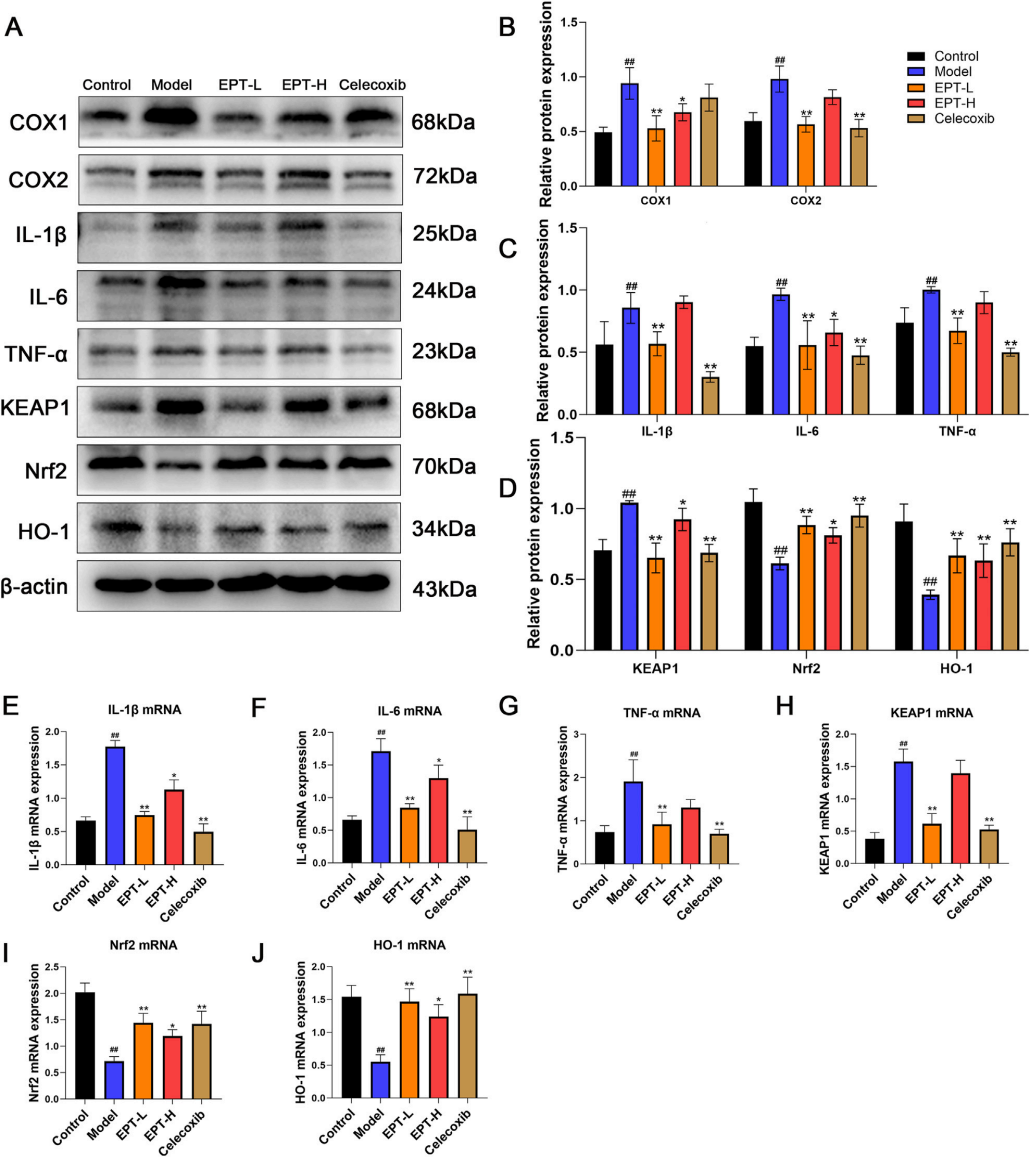
The improvement of inflammation and oxidative stress in NAFLD rats by EPT. **(A–D)** Western blot analysis of COX1, COX2, IL-1β, IL-6, TNF-α, KEAP1, Nrf2, and HO-1 protein expression levels in rat liver tissue. **(E–J)** RT-qPCR analysis of the relative mRNA transcription levels of IL-1β, IL-6, TNF-α, KEAP1, Nrf2, and HO-1 in rat liver tissue. These results were presented as mean ± SD (All detections were performed on the three optimal samples selected following three independent experiments, with n = 3).

As COX2 is a critical enzyme in arachidonic acid metabolism that produces inflammatory mediators, we assessed the expression of inflammation-related factors (COX1, COX2, IL-1β, IL-6, TNF-α) and oxidative stress-related proteins (KEAP1, Nrf2, HO-1) in liver tissue using Western blot analysis. The model group showed significantly increased levels of COX1, COX2, IL-1β, IL-6, TNF-α, KEAP1, and accompanied by decreased levels of Nrf2, HO-1,. EPT treatment significantly reversed these changes (Figures 6A–D). Consistent results were observed for the mRNA expression of these proteins (Figures 6E–J), indicating that EPT might ameliorate inflammation and oxidative stress by inhibiting COX2 expression.

### 3.7 EPT protects against hepatic ER stress in NAFLD model rats

**FIGURE 7:**
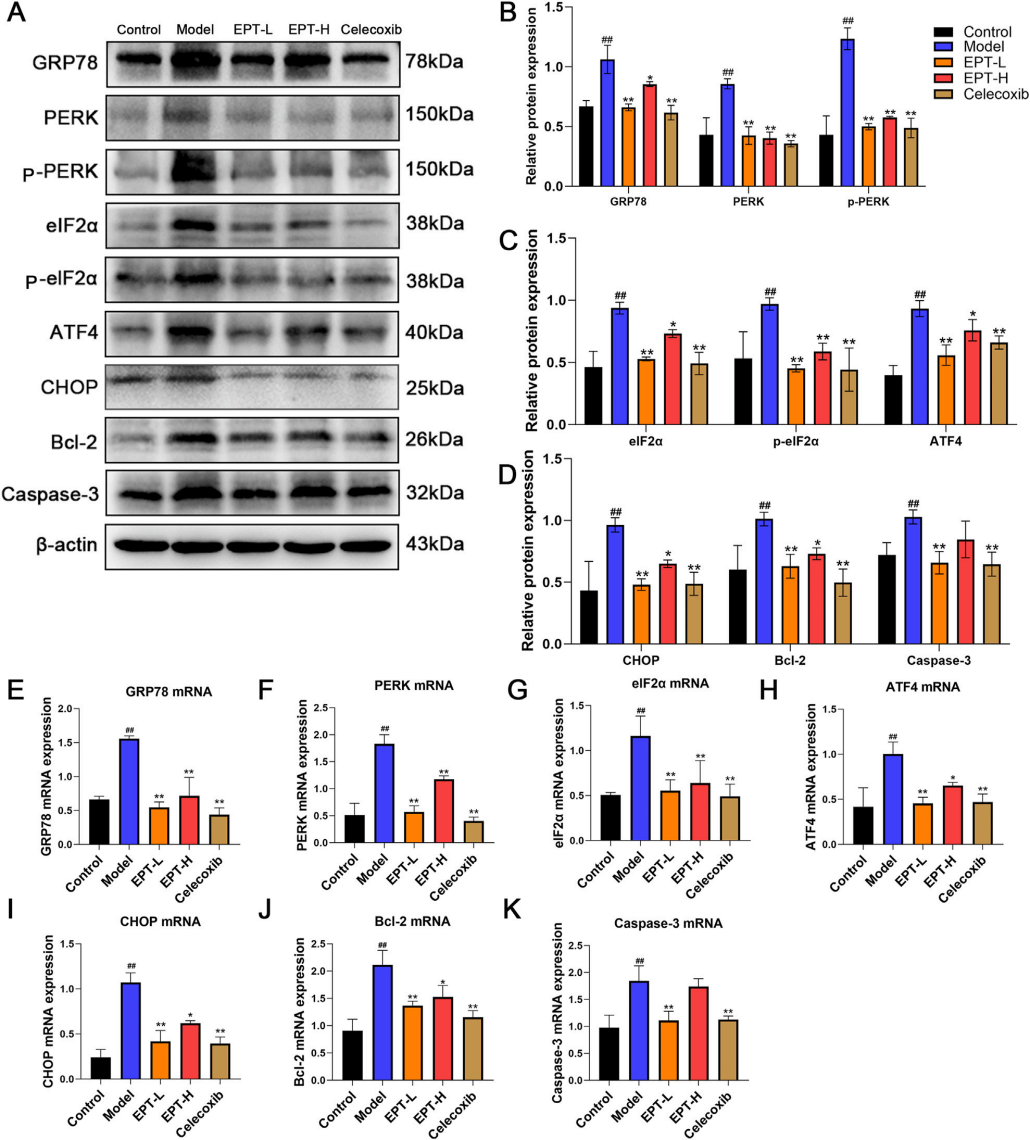
The effects of EPT on the PERK pathway of ER stress and its downstream proteins and mRNA. **(A–D)** Western blot analysis of GRP78, PERK, p-PERK, eIF2α, p-eIF2α, ATF4, CHOP, Bcl-2, and Caspase-3 protein expression levels in rat liver tissue. **(E–K)** RT-qPCR analysis of the relative mRNA transcription levels of GRP78, PERK, eIF2α, ATF4, CHOP, Bcl-2, and Caspase-3 in rat liver tissue. These results were presented as mean ± SD (n = 3). ^##^p < 0.01 compared with the control group. *p < 0.05, **p < 0.01 compared with the Model group.

To investigate the relationship between EPT and ER stress, we examined the PERK pathway and its downstream targets using Western blot analysis. In the model group, the expression of GRP78, PERK, phosphorylated PERK (p-PERK), eIF2α, phosphorylated eIF2α (p-eIF2α), ATF4, CHOP, Bcl-2, and Caspase-3 was significantly elevated. EPT treatment markedly reduced these expression levels (Figures 7A–D). The corresponding mRNA expression patterns were consistent with the protein levels (Figures 7E–K), demonstrating that EPT might alleviate ER stress in NAFLD rats via the PERK-eIF2α-ATF4 pathway and simultaneously inhibits apoptosis.

**FIGURE 8:**
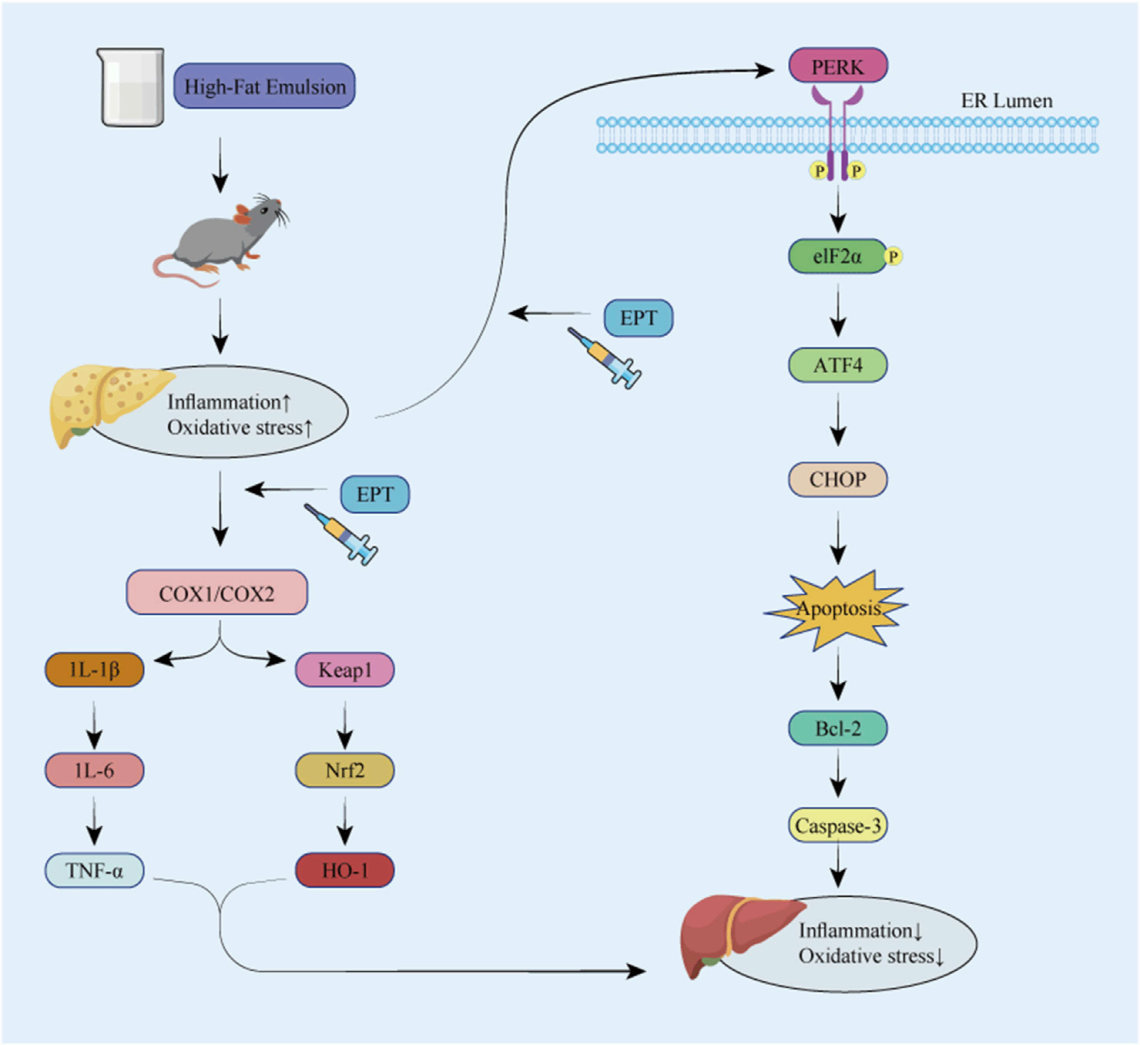
Overall mechanism diagram of the experiment.

## 4 Discussion

This study identified the blood-absorbed components of EPT when taken orally and investigated its mechanisms in delaying the progression of NAFLD. Using UPLC-MS/MS technology, we analyzed the components absorbed into the serum of rats treated with EPT. Through network pharmacology and transcriptomics approaches, we screened the main targets and signaling pathways associated with EPT’s therapeutic effects. Further experiments show that EPT can significantly inhibit COX2 to reduce inflammation and oxidative stress, and regulate the PERK - ELF2α - ATF4 pathway to alleviate ER stress and apoptosis. These findings help us preliminarily explore EPT’s early molecular mechanisms in delaying NAFLD progression, offering initial evidence for its potential application in NAFLD treatment.

Traditional Chinese medicine-derived compounds that enter the bloodstream play a critical role in disease treatment. Using UPLC-MS/MS, we identified 456 blood-absorbed compounds in the serum of rats treated with EPT, including saponins, flavonoids, alkaloids, terpenoids, lignans, coumarins, phenolic acids, quinones, and other substances. Representative compounds such as Fallaxsaponin A, Tenuifoliside B, and Polygalaxanthone IX exhibit notable anti-inflammatory and anti-apoptotic effects (Dong et al., 2014). Tenuifolin and Polygalaxanthone V scavenge free radicals, reduce pro-inflammatory factor production, and exhibit anti-inflammatory and antioxidant properties (Jiang et al., 2023; Liu et al., 2021). Similarly, Tenuifoliside A and Presenegenin present with strong anti-inflammatory potential. These findings suggest that the components of EPT that are ultimately absorbed into the blood possess excellent anti-inflammatory and antioxidant characteristics (Kim et al., 2013; Luyen et al., 2024). Network pharmacology analysis further indicated that EPT influences fatty acid metabolism, reduces lipid accumulation, and regulates pathways such as arachidonic acid metabolism, NF-κB signaling, TNF signaling, IL-17 signaling, AMPK signaling, and ferroptosis. These effects ultimately enhance antioxidant defenses, improve mitochondrial function, and mitigate oxidative stress. The observed enrichment in ER-associated and apoptosis-related pathways suggests that EPT might protect hepatocytes by alleviating oxidative damage. Together, these findings suggest that EPT might exert significant therapeutic effects in the treatment of NAFLD by regulating lipid metabolism, inhibiting inflammatory responses, alleviating oxidative damage, and improving stress conditions, thereby preventing protein misfolding.

In the progression of NAFLD, patients commonly exhibit hepatic fat accumulation, hepatocyte degeneration, and abnormal liver function, alongside chronic inflammation and gradual fibrosis (Han et al., 2023; Wang et al., 2024). Clinical indicators include elevated TC, TG, AST, and ALT levels, with histopathological features such as lipid vacuoles, fat deposition, and collagen accumulation (Pafili and Roden, 2021; Lu and Wang, 2025; Li et al., 2025). Consistent with this, the present analyses revealed the yellowing of the liver, hyperlipidemia, and liver injury in the model group rats. H&E, Oil Red O, and Sirius Red staining revealed significant lipid accumulation, fat deposition, and collagen accumulation in the model group’s liver tissues. However, EPT treatment significantly reversed these pathological changes, indicating that EPT effectively reduces lipid accumulation, alleviates liver injury, and mitigates NAFLD.

Transcriptomic sequencing performed based on these initial analyses provided further insights into the underlying mechanisms through which EPT exerts its beneficial effects. Significant changes were observed in the arachidonic acid metabolism pathway, inflammation-related pathways, and ER-related pathways after EPT treatment, aligning with network pharmacology predictions. Our transcriptomic analysis identified key targets like PTGS2 (COX-2) and Keap1. EPT was found to markedly downregulate PTGS2, suggesting that it might reduce prostaglandin synthesis and, consequently, alleviate inflammation. Keap1 modulation suggests EPT might activate the Keap1 - Nrf2 pathway to curb oxidative stress (Li et al., 2024; Yang et al., 2024). Additionally, some differentially expressed genes were enriched in ER - associated pathways, indicating that the pharmacological effects of EPT are linked to ER stress modulation. In this experiment. Based on targeting COX2 and modulating the Keap1 - Nrf2 and ER stress - related pathways, we preliminarily explored the underlying mechanism of EPT in alleviating early NAFLD progression.

COX2 is the rate - limiting enzyme in arachidonic acid metabolism. It generates prostaglandins and other bioactive lipid mediators that play crucial roles in inflammation and metabolic processes (Ilari et al., 2020). In NAFLD, COX2 expression is significantly upregulated and closely linked to hepatic steatosis and inflammation (Desai et al., 2018). This upregulation induces the release of pro - inflammatory cytokines such as IL - 1β, IL - 6, and TNF - α, thereby exacerbating inflammation and driving the pathological progression of NAFLD (Cheng et al., 2013). Moreover, COX2 overexpression is associated with oxidative stress and lipid metabolic disorders, which are key mechanisms in NAFLD development (Tsujimoto et al., 2016). In rats with high-fat-diet-induced NASH, celecoxib, a selective COX2 inhibitor, significantly reduces hepatic lipid accumulation and improves insulin resistance (Tian et al., 2014). Additionally, celecoxib can also mitigate high-fat-diet-induced steatosis and inflammation in NASH model rats (Chen et al., 2011). NASH and NAFLD share similar pathological features in the early stages of disease progression. Additionally, PGE2, a downstream product of COX2, acts via its receptors in the liver. It is involved in inflammatory responses and lipid metabolism (Wang et al., 2021). These clues indicate that COX2 might be a potential target for early NAFLD prevention and treatment. Our prior research shows that EPT can effectively suppress PGE2 expression, offering anti-inflammatory effects (Wen et al., 2015). Thus, in this study, we chose COX2 as the target to preliminarily explore the molecular mechanism of EPT in preventing and treating NAFLD.

In NAFLD research, Keap1, a key oxidative stress regulator, has been widely studied for its mechanism and potential as a therapeutic target. Keap1 interacts with Nrf2 to regulate cellular antioxidant responses and metabolic homeostasis. Under normal conditions, Keap1 inhibits Nrf2, but under oxidative stress, they detach, allowing Nrf2 to move to the nucleus. Studies have shown that inflammation and oxidative stress are crucial factors in the development of liver diseases, including NAFLD. Nrf2, a key regulator of cellular protection, defends the liver through its anti-inflammatory and antioxidant functions. The activation or inhibition of Nrf2 can significantly impact the progression of such diseases (Xu et al., 2018). Moreover, the Keap1-Nrf2 pathway in NAFLD exerts anti-inflammatory, antioxidant, and lipid - metabolism regulatory effects and also protects hepatocytes from lipotoxic injury by promoting autophagy (Lee et al., 2020; Gao et al., 2021). Thus, targeting the Keap1 - Nrf2 pathway might be a potential therapeutic strategy for NAFLD. HO-1, a downstream protein of the Keap1-Nrf2 pathway, offers hepatic protection through anti-inflammatory, antioxidant, and cytoprotective mechanisms. Studies have shown that HO-1 downregulation is closely related to inflammation, oxidative stress, and hepatocyte injury during NAFLD progression. Enhancing HO-1 expression might be an effective strategy for treating liver diseases (Li et al., 2023). In this study, rats in the model group showed significantly increased Keap1 expression and decreased Nrf2 and HO-1 expression, indicating weakened anti-inflammatory and antioxidant capacity in NAFLD rats. After EPT administration, we found that EPT significantly reduced Keap1 expression and increased Nrf2 and HO-1 expression. This suggests that EPT can restore the anti-inflammatory and antioxidant capacity in NAFLD rats, thereby improving NAFLD.

Inflammation and oxidative stress exacerbate ERS, ERS reciprocally intensifies inflammation and oxidative stress, this interplay complicates the progression of NAFLD (Malhotra and Kaufman, 2011). The endoplasmic reticulum (ER) is responsible for protein processing, folding, and modification. During inflammation, COX2 upregulation increases prostaglandin synthesis, inducing the release of pro - inflammatory cytokines such as IL - 1β, IL - 6, and TNF - α. This disrupts ER function, leads to increased protein translation, and results in the accumulation of misfolded proteins (Shieh et al., 2024; Yu and Kim, 2010). Simultaneously, the COX2 - catalyzed arachidonic acid metabolism generates substantial ROS. This causes oxidative stress, disrupts the ER’s redox balance, intensifies protein folding issues, and exceeds the ER’s processing capacity, thereby inducing ERS. ERS is closely related to hepatic metabolic functions. In metabolic syndrome contexts like obesity and type 2 diabetes, sustained ERS activation causes intrahepatic lipid and cholesterol accumulation. This triggers oxidative stress, further intensifying ERS (Bozaykut et al., 2016). In the pathophysiology of NAFLD, ERS is closely linked to autophagy dysfunction. Autophagy, a cellular degradation process that removes damaged organelles and proteins, is inhibited by ERS activation. This inhibition exacerbates hepatic lipid accumulation and inflammation, promoting the progression of NAFLD to NASH (Lebeaupin et al., 2018). Moreover, in NAFLD, ERS affects not only hepatocytes but also other liver cells like hepatic stellate cells (HSCs) and Kupffer cells (KCs). During NAFLD progression to NASH, activated HSCs produce excessive extracellular matrix, causing liver fibrosis and releasing cytokines to worsen inflammation. Kupffer cells exacerbate hepatic inflammation by releasing pro - inflammatory cytokines and participating in lipid metabolism regulation. Simultaneously, ERS also intensifies inflammation and oxidative stress, creating a positive feedback loop that accelerates NAFLD progression toward NASH (Flessa et al., 2022). These findings suggest that exploring the relationship between ERS and NAFLD might perhaps offer new therapeutic insights and potential targets for NAFLD treatment.

When ERS is persistently activated, the body triggers the unfolded protein response (UPR) to restore ER homeostasis. Initially, this process activates the PERK pathway. Upon dissociation from GRP78, PERK dimerizes and undergoes trans-autophosphorylation, activating its kinase domain. This activation further phosphorylates the α-subunit of eukaryotic translation initiation factor 2 (eIF2α), halting the translation of most mRNAs to alleviate ERS (Liu et al., 2015). However, it simultaneously enhances the translation of activating transcription factor 4 (ATF4), selectively upregulating the expression of C/EBP homologous protein (CHOP). The upregulation of CHOP, in coordination with Bcl-2 expression, regulates apoptotic signaling pathways and activates the apoptotic effector protein Caspase-3, further promoting the execution of apoptosis (Zinszner et al., 1998). In this study, model group rats exhibited significant increases in GRP78, PERK, p-PERK, eIF2α, p-eIF2α, ATF4, CHOP, Bcl-2, and Caspase-3 expression, indicating pronounced ERS and apoptosis. It demonstrates significant ER stress and apoptosis. After EPT treatment, the significant reduction in these indicators suggests that EPT might alleviate ER stress and inhibit apoptosis by modulating the PERK-ELF2α-ATF4 pathway.

Our experiment in this study also has certain limitations. In our previous study, we demonstrated EPT’s effect on PGE2. The primary objective of this experiment was to observe whether EPT could act as a selective COX2 inhibitor to improve NAFLD. Consequently, we used celecoxib, a selective COX2 inhibitor, as a positive control, rather than clinically established NAFLD drugs like pioglitazone. This is a limitation of our study. In this experiment, based on the transcriptomic results, we also made a preliminary exploration of the ER stress pathway. The results indicate that EPT has a good inhibitory effect on ER stress. However, the failure to select an inhibitor with blocking effects on ER stress as a positive control is also a limitation of this experiment. Additionally, NAFLD is a multifactorial disease involving numerous signaling cascades. This raises concerns that a single signaling pathway might not fully elucidate a drug’s mechanism of action in treating NAFLD. In this experiment, we only explored the COX2 and PERK-eIF2α-ATF4 pathways. We failed to show broader metabolic evidence and explore other potential therapeutic pathways for NAFLD. These are also limitations of this experiment. In follow-up experiments, we will strive to improve these shortcomings by enhancing experimental clarity, transparency, and scientific soundness.

## 5 Conclusion

In summary, our exploration of NAFLD’s early molecular mechanisms indicates that EPT might target COX2 to exert anti-inflammatory and antioxidant effects. It might also alleviate ER stress and inhibit apoptosis via the PERK-ELF2α-ATF4 pathway, thereby improving NAFLD. In subsequent studies, we will further explore the correlation between the COX2 and PERK-ELF2α-ATF4 pathways.

## Data Availability

The data presented in the study are deposited in the NCBI (SRA) repository, accession number PRJNA1271756. Available at: https://www.ncbi.nlm.nih.gov/sra/PRJNA1271756.

## Abbreviations

NAFLD, Non-alcoholic fatty liver disease; EPT, *Polygala tenuifolia* Willd. Extract; ALT, Alanine aminotransferase; AST, Aspartate aminotransferase; IL-1β, Interleukin-1β; IL-6, Interleukin-6; TNF-α, Tumor necrosis factor-α; NF-κB, Nuclear factor-kappa B; KEAP1, Kelch-like ECH-associated protein 1; Nrf2, Nuclear factor erythroid 2-related factor 2; HO-1, Heme oxygenase-1; GRP78, 78 kDa glucose-regulated protein; PERK, Protein kinase R-like endoplasmic reticulum kinase; elF2α, Eukaryotic translation initiation factor 2; ATF4, Activating transcription factor 4; CHOP, C/EBP homologous protein; Bcl-2, B-cell lymphoma-2; Caspase-3, cysteine protease protein 32kD.
