# Supplementary figures and images for: Polygala tenuifolia Willd. Extract delays non-alcoholic fatty liver disease progression in rats via the COX2 and PERK-elF2α-ATF4 pathway
Source: Front Pharmacol. 2025 Jun 12;16:1595752. doi: 10.3389/fphar.2025.1595752 (PMC12198203; doi:10.3389/fphar.2025.1595752)

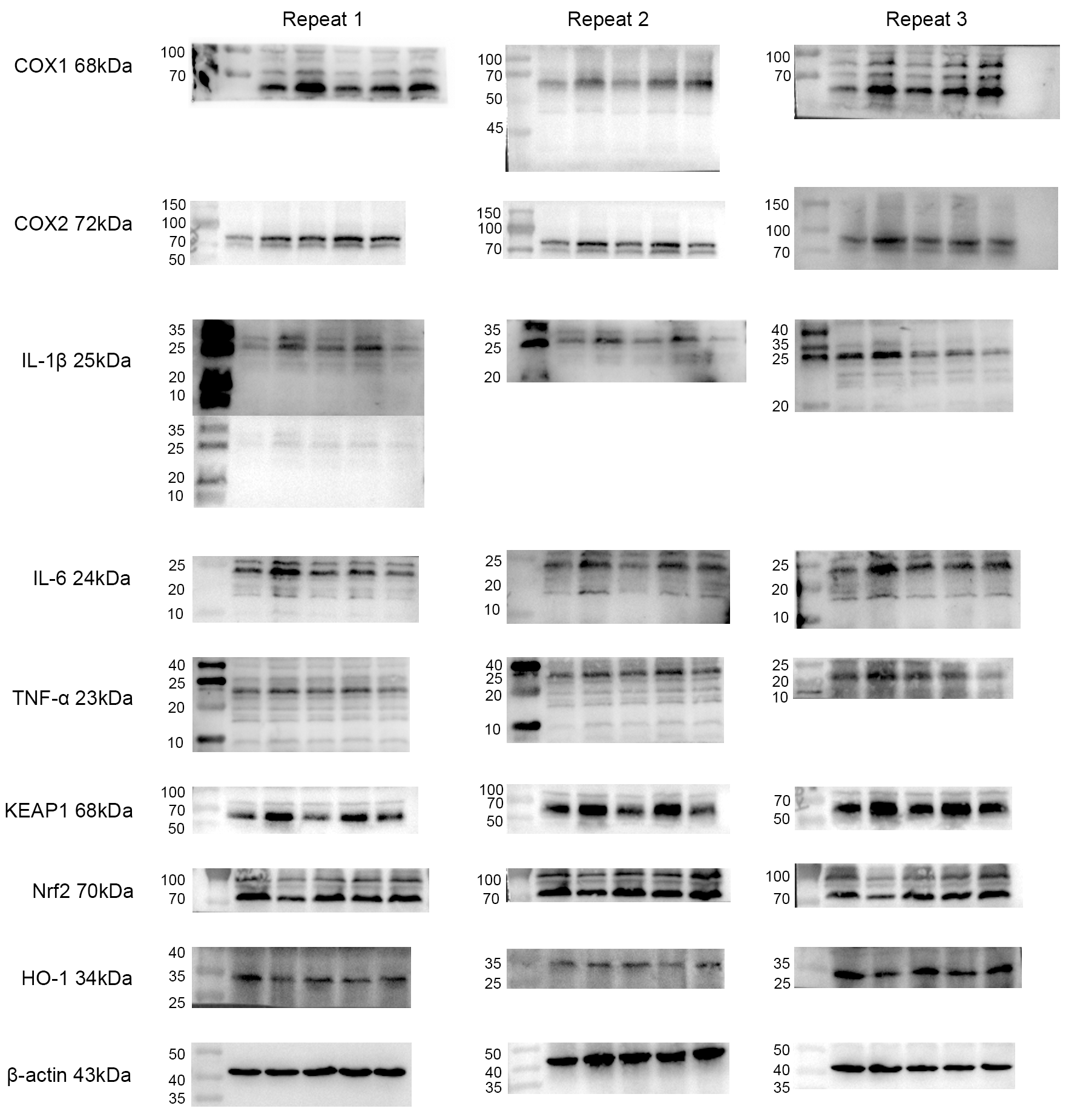

Supplement: Supplementary file 1 [file Image3.TIF]

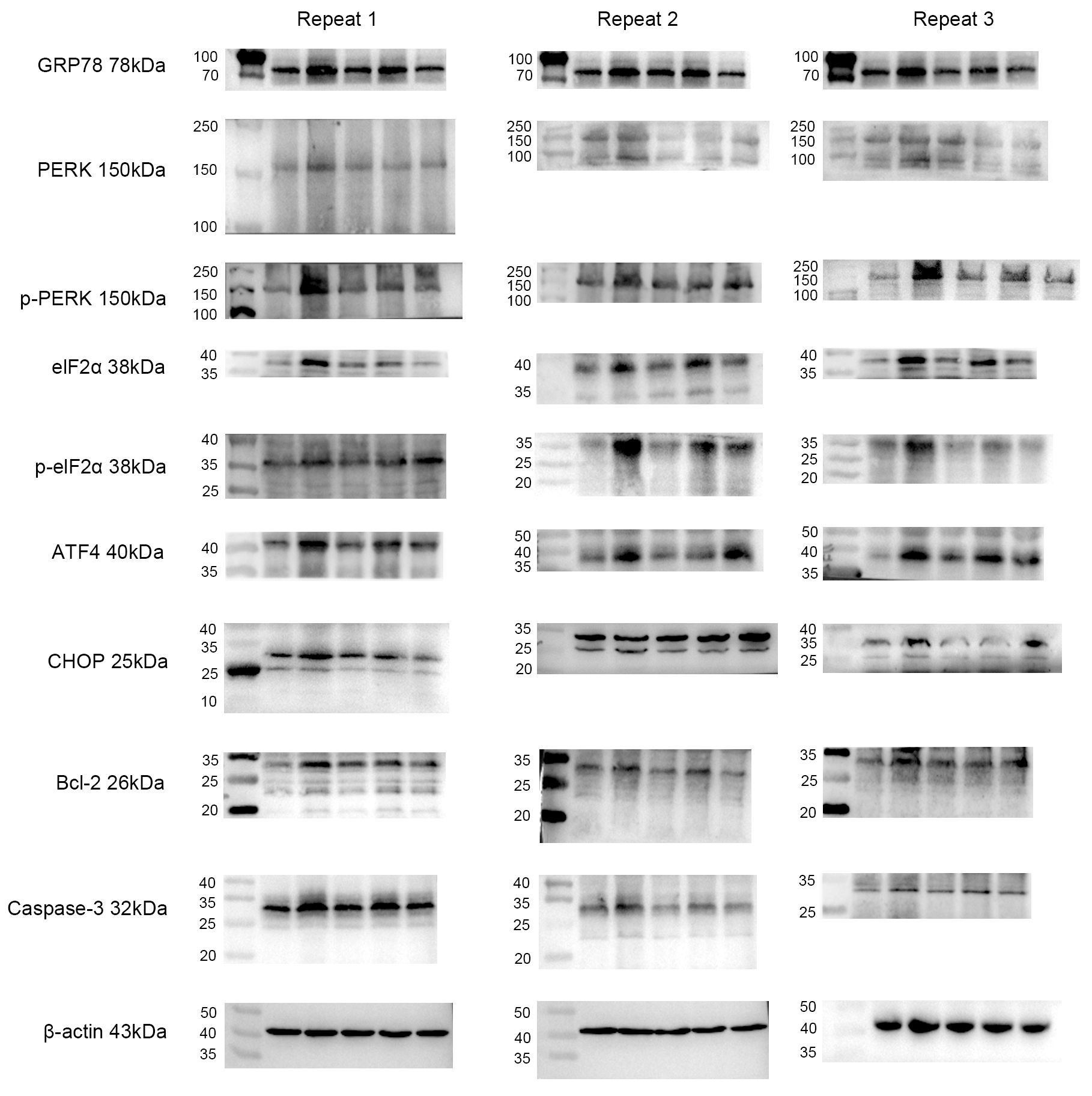

Supplement: Supplementary file 2 [file Image4.TIF]

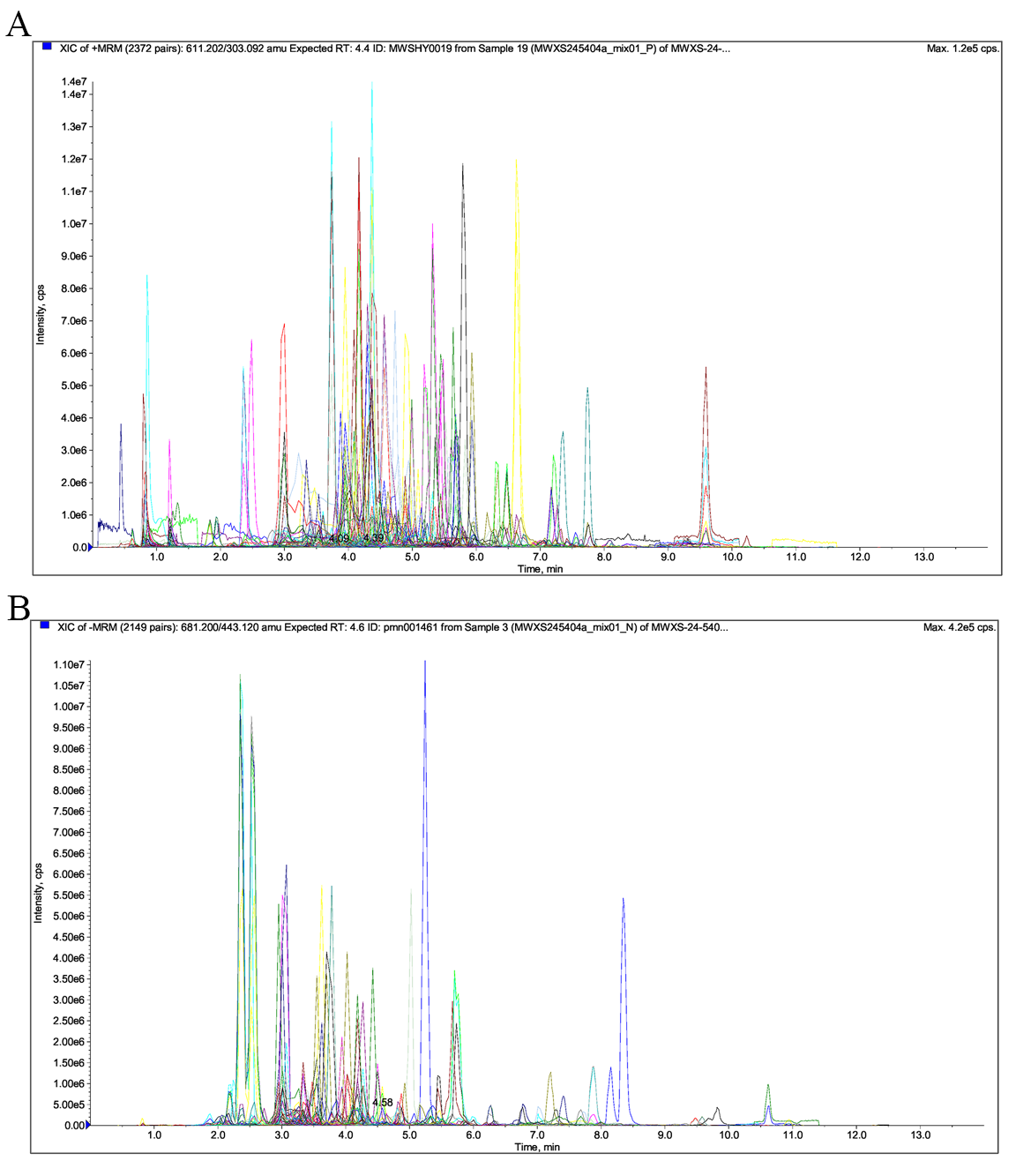

Supplement: Supplementary file 3 [file Image2.TIF]

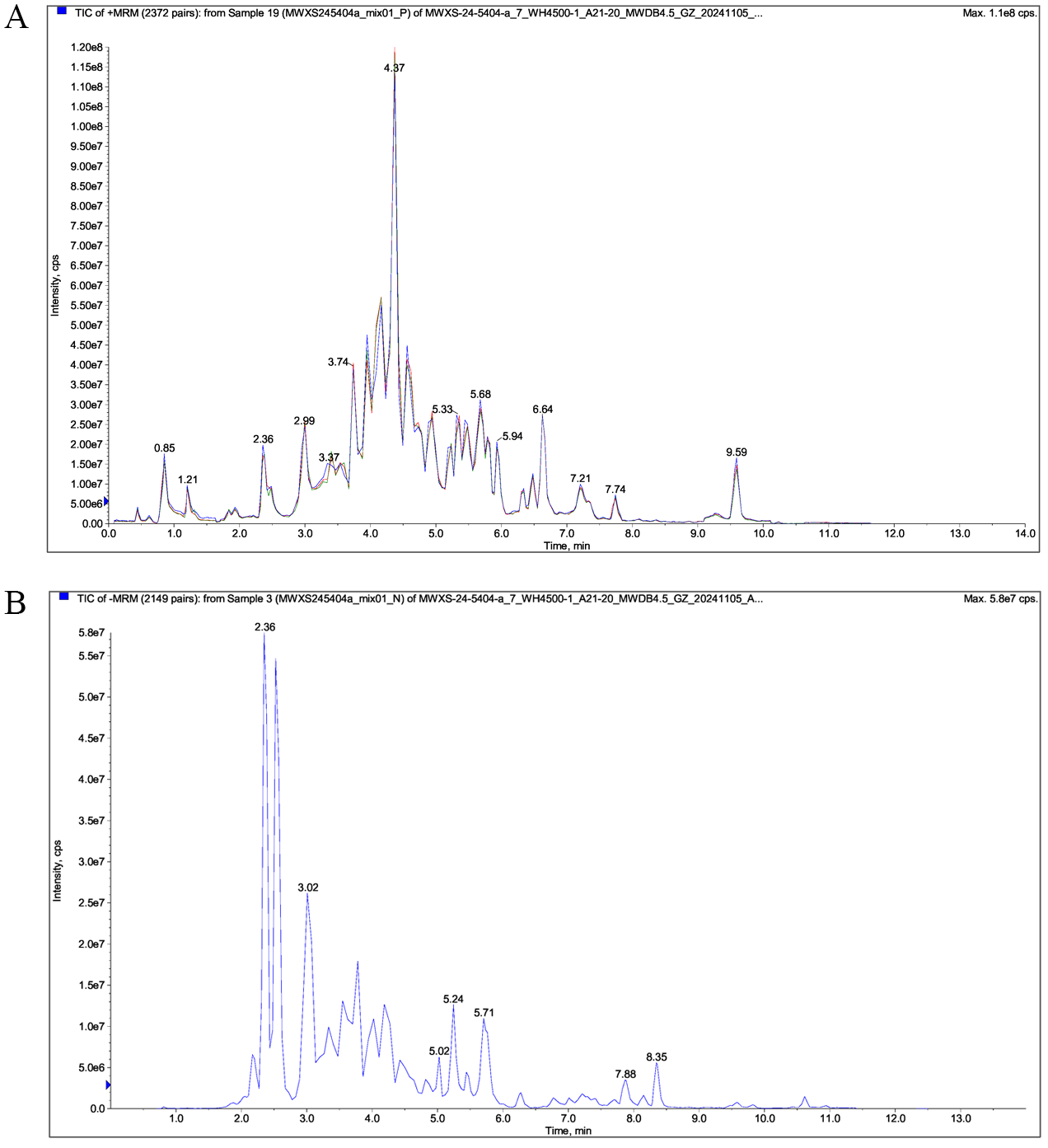

Supplement: Supplementary file 4 [file Image1.TIF]
