# Supplementary material for: Polygala tenuifolia Willd. Extract delays non-alcoholic fatty liver disease progression in rats via the COX2 and PERK-elF2α-ATF4 pathway
Source: Front Pharmacol. 2025 Jun 12;16:1595752. doi: 10.3389/fphar.2025.1595752 (PMC12198203; doi:10.3389/fphar.2025.1595752)
Supplement: Supplementary file 5 [file DataSheet1.docx]

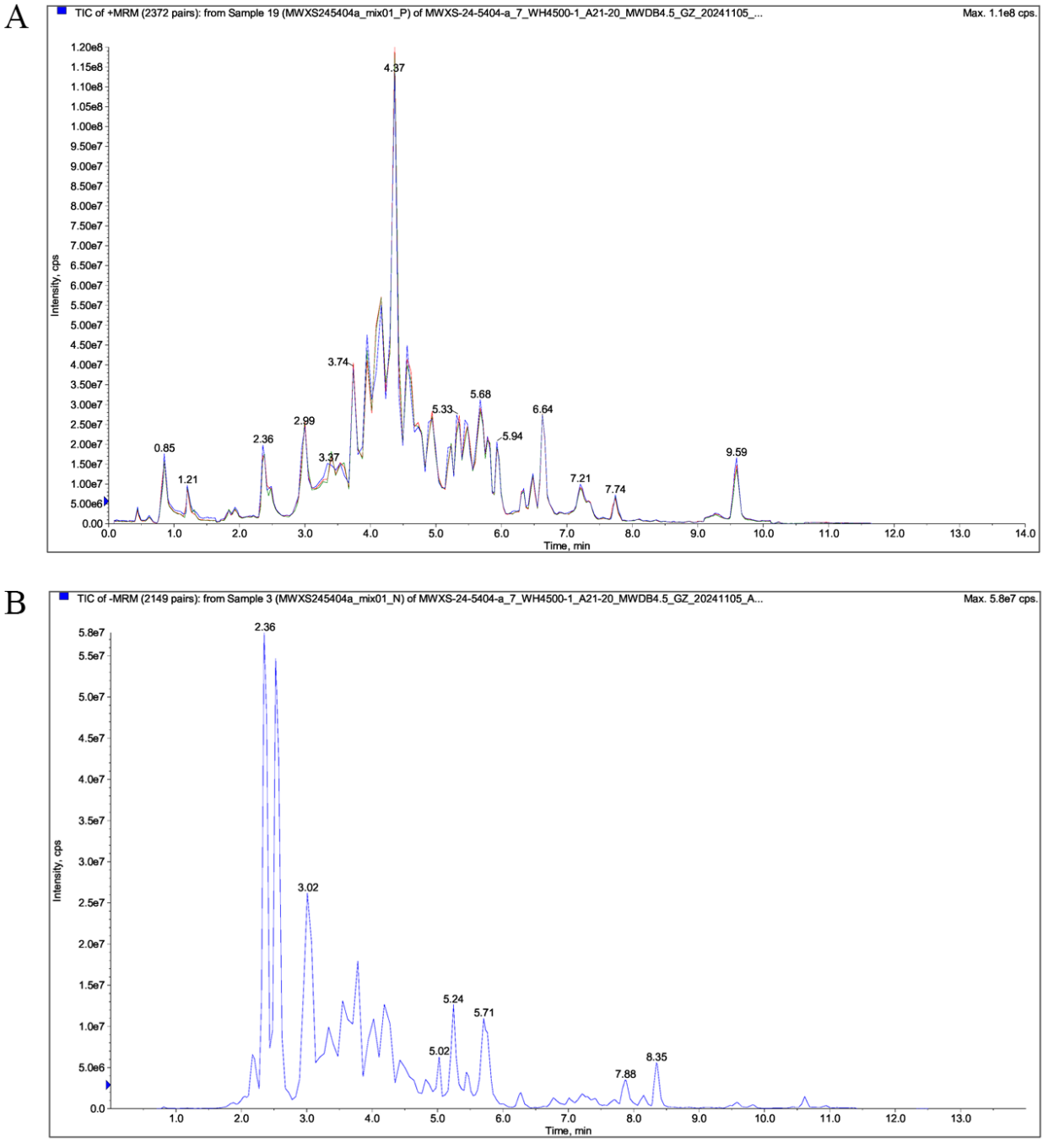


**Supplementary Figure 1.**The total ion chromatogram under positive and negative ionization modes. (A) Total ion current diagram in positive ion mode. (B) Total ion current diagram in negative ion mode.


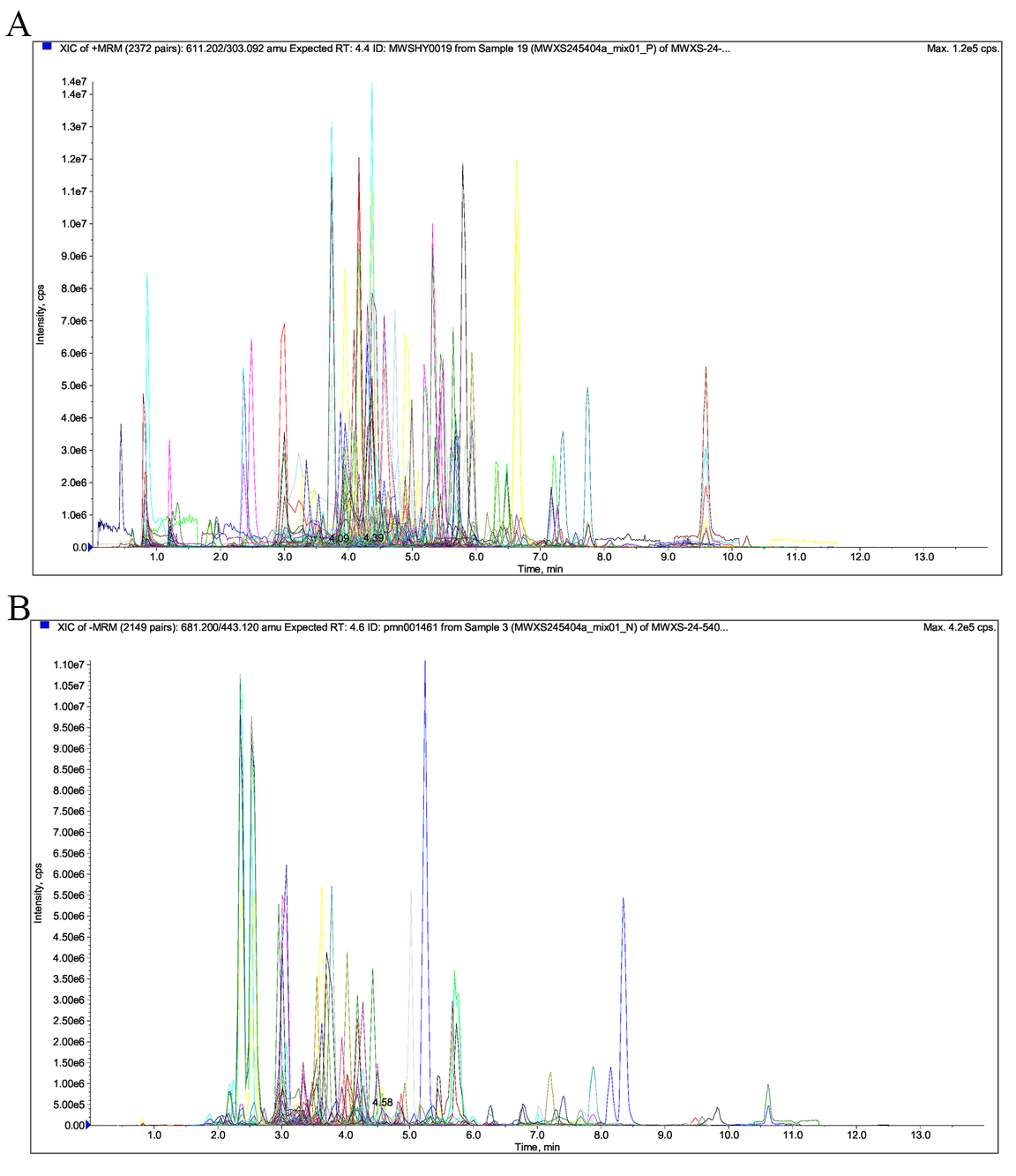


**Supplementary Figure 2.**The MRM chromatogram under positive and negative ionization modes. (A) MRM chromatogram in positive ion mode. (B) MRM chromatogram in negative ion mode.


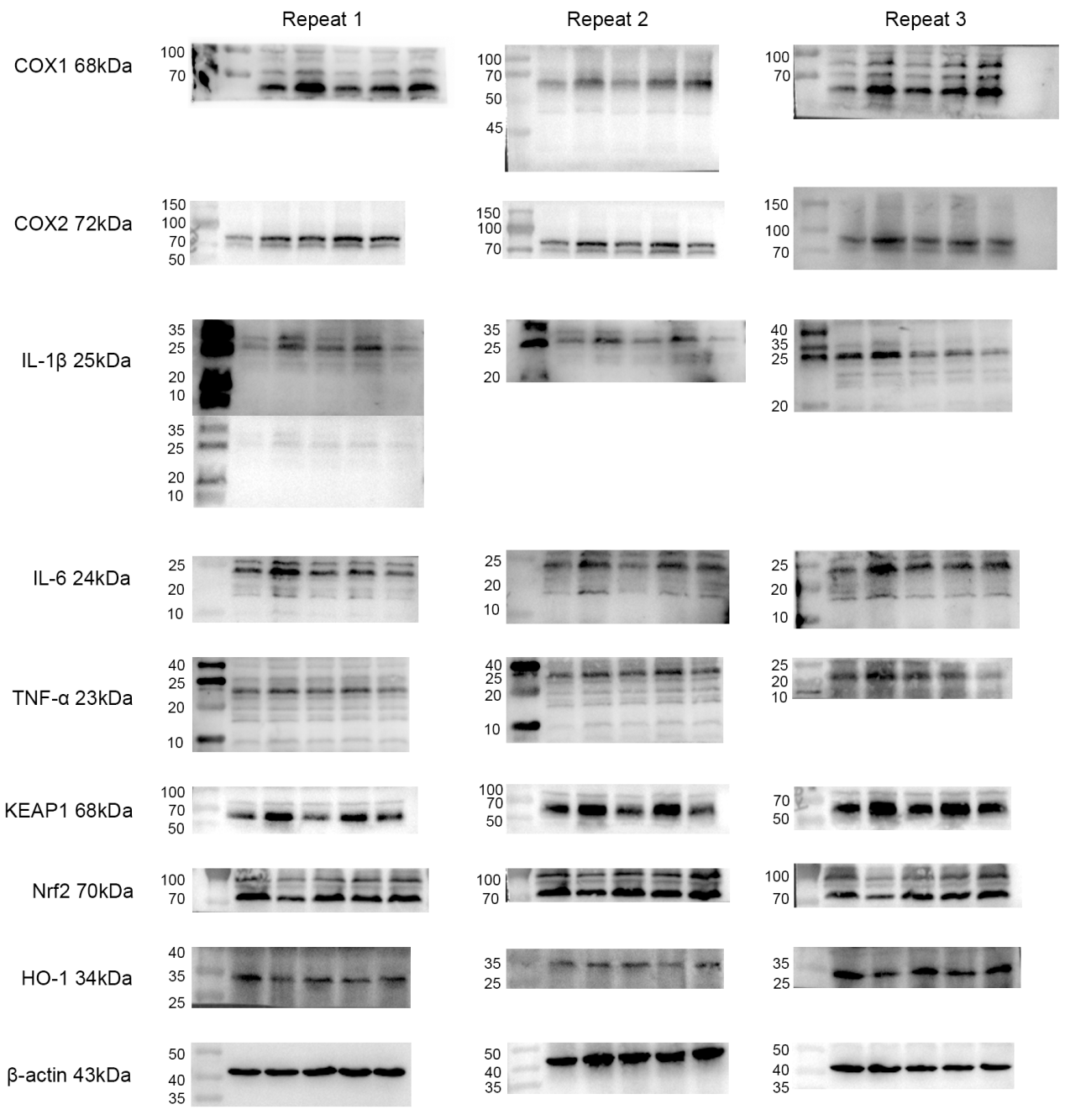


**Supplementary Figure 3.**Triple replicate images of Western blot in Fig6


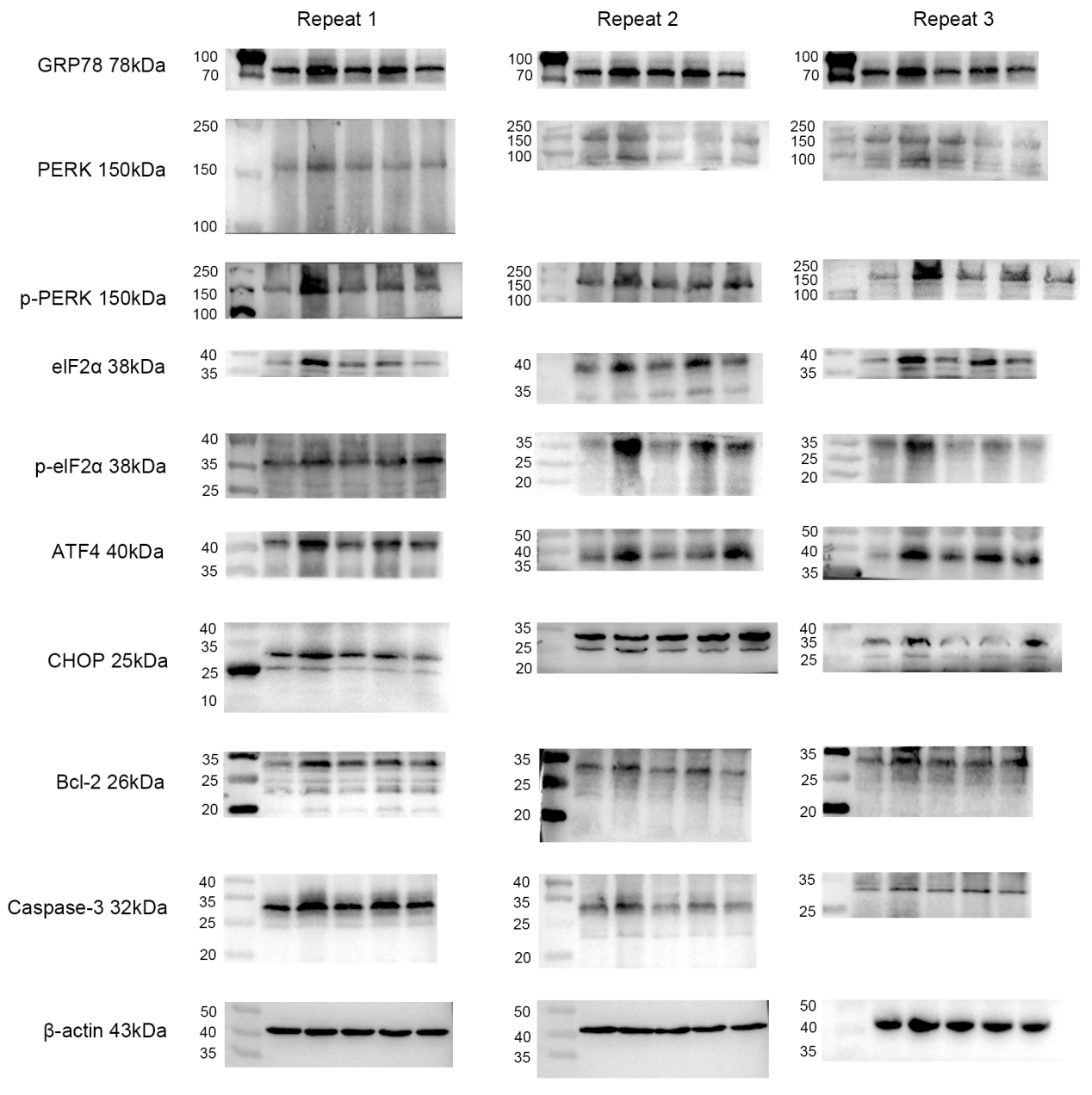


**Supplementary Figure 4.**Triple replicate images of Western blot in Fig7

**Supplementary Table 1**

Identification of EPT Blood Components.

|  | Compounds | Formula |
| --- | --- | --- |
| 1 | Tenuifoliside A | C_31_H_38_O_17_ |
| 2 | 3,4,5-Trimethoxycinnamic acid | C_12_H_14_O_5_ |
| 3 | Arillanin A | C_33_H_40_O_18_ |
| 4 | 2,5-O-Di-CinnamylSorbitol | C_24_H_30_O_6_ |
| 5 | Bis(2-ethyl-hexyl)-phthalate | C_24_H_38_O_4_ |
| 6 | Diisooctyl Phthalate | C_24_H_38_O_4_ |
| 7 | Dibutyl phthalate | C_16_H_22_O_4_ |
| 8 | Diisobutyl phthalate | C_16_H_22_O_4_ |
| 9 | Butyl isobutyl phthalate | C_16_H_22_O_4_ |
| 10 | 3,3'-Oxydipropyl dibenzoate | C_20_H_22_O_5_ |
| 11 | Bis(_2_-ethylhexyl)phthalate | C_24_H_38_O_4_ |
| 12 | Anthranilate | C_7_H_7_NO_2_ |
| 13 | Tenuifoliside D | C_18_H_24_O_9_ |
| 14 | 4-Coumarate | C_9_H_8_O_3_ |
| 15 | Cinnamaldehyde | C_9_H_8_O |
| 16 | 1,7-bis(4-hydroxypheny)hept-_1_-ene-_3_-ol | C_19_H_22_O_3_ |
| 17 | Trollioside | C_19_H_26_O_9_ |
| 18 | Eudesmic acid (3,4,5-trimethoxybenzoic acid) | C_10_H_12_O_5_ |
| 19 | 3-O-(p-coumaroyl) 3-Hydroxy-3-methylglutaric acid | C_15_H_16_O_7_ |
| 20 | Ningposide A | C_18_H_22_O_9_ |
| 21 | Methyl-3-(3-hydroxyphenyl)Propionate | C_10_H_12_O_3_ |
| 22 | (3,4,5-trihydroxytetrahydro-2H-pyran-2-yl)methyl (E)-3-(4-hydroxy-3-methoxyphenyl)acrylate | C_16_H_18_O_8_ |
| 23 | Sibiricose A1 | C_34_H_42_O_19_ |
| 24 | 1-O-(p-coumaroyl) 3-Hydroxy-3-methylglutaric acid | C_15_H_16_O_7_ |
| 25 | 2-O-Galloyl-D-glucose | C_13_H_16_O_10_ |
| 26 | 2,4-Di-Tert-Butylphenol | C_14_H_22_O |
| 27 | 2,3-bis(2-carboxyphenyl)succinic acid | C_18_H_14_O_8_ |
| 28 | Piperonylic acid | C_8_H_6_O_4_ |
| 29 | Ferulate | C_10_H_10_O_4_ |
| 30 | 2-O-Galloylmucic acid Dimethyl Ester | C_15_H_18_O_12_ |
| 31 | Terephthalate | C_8_H_6_O_4_ |
| 32 | 3-(4-Hydroxyphenyl)lactate | C_9_H_10_O_4_ |
| 33 | Methyl 2,4-dihydroxyphenylacetate | C_9_H_10_O_4_ |
| 34 | trans-2-Hydroxycinnamate | C_9_H_8_O_3_ |
| 35 | Isoferulic Acid | C_10_H_10_O_4_ |
| 36 | Stypandrol | C_26_H_22_O_6_ |
| 37 | Caffeate | C_9_H_8_O_4_ |
| 38 | 3-(Methylferuloyl)-6'-p-hydroxybenzoyl-sucrose | C_30_H_36_O_16_ |
| 39 | Phenylpropanoate | C_9_H_10_O_2_ |
| 40 | 6-O-Feruloyl-glucose | C_16_H_20_O_9_ |
| 41 | Phloretate | C_9_H_10_O_3_ |
| 42 | Phenyllactate | C_9_H_10_O_3_ |
| 43 | 3-Methylferuloyl-6'-(3-hydroxy-p-p-coumaroyl)-sucrose | C_30_H_36_O_17_ |
| 44 | 3-O-Galloyl-β-D-Glucose | C_13_H_16_O_10_ |
| 45 | DL-3-Phenyllactic acid | C_9_H_10_O_3_ |
| 46 | trans-Cinnamate | C_9_H_8_O_2_ |
| 47 | 3-Serpinoyl-6'-(4-Methylspinacyl)-sucrose | C_35_H_44_O_19_ |
| 48 | Tenuifoliside C | C_35_H_44_O_19_ |
| 49 | Formylanthranilate | C_8_H_7_NO_3_ |
| 50 | Syringic acid | C_9_H_10_O_5_ |
| 51 | 3-Methylspinacyl-6'-benzoyl-sucrose | C_31_H_38_O_16_ |
| 52 | 3-Methylferuloyl-6'-(3-hydroxy-feruloyl)-sucrose | C_33_H_40_O_18_ |
| 53 | 3,6'-Disinapoylsucrose | C_34_H_42_O_19_ |
| 54 | 3-Hydroxyphenylacetic Acid Methyl Ester | C_9_H_10_O_3_ |
| 55 | Ferulic acid methyl ester | C_11_H_12_O_4_ |
| 56 | 4-O-Glucosyl-4-hydroxybenzoic acid | C_13_H_16_O_8_ |
| 57 | 4,5-dihydroxy-6-((3-hydroxy-4-((3-(4-hydroxy-3,5-dimethoxyphenyl)acryloyl)oxy)-2,5-bis(hydroxymethyl)tetrahydrofuran-2-yl)oxy)-2-(hydroxymethyl)tetrahydro-2H-pyran-3-yl benzoate | C_30_H_36_O_16_ |
| 58 | 3-Spinoyl-6'-benzoyl-sucrose | C_30_H_36_O_16_ |
| 59 | reinoisoe B* | C_29_H_34_O_15_ |
| 60 | 3-Feruloyl-6'-benzoyl-sucrose | C_29_H_34_O_15_ |
| 61 | 2,4-Dihydroxybenzoic acid | C_7_H_6_O_4_ |
| 62 | 1-O-p-Cumaroylglycerol | C_12_H_14_O_5_ |
| 63 | Sinapoyl aldehyde | C_11_H_12_O_4_ |
| 64 | 3-Methoxybenzoic acid | C_8_H_8_O_3_ |
| 65 | 3,4-Dihydroxybenzoate | C_7_H_6_O_4_ |
| 66 | 4-O-beta-D-Glucosyl-sinapate | C_17_H_22_O_10_ |
| 67 | 6-{[(2s,3r,4s,5s,6r)-4,5-dihydroxy-6-(hydroxymethyl)-3-{[(2s,3r,4r,5r,6s)-3,4,5-trihydroxy-6-methyloxan-2-yl]oxy}oxan-2-yl]oxy}-1,3-dihydroxy-7-methoxyxanthen-9-one | C_26_H_30_O_15_ |
| 68 | 7-Methoxy-3-[1-(3-pyridyl)methylidene]-4-chromanone | C_16_H_13_NO_3_ |
| 69 | Scuteamoenoside | C_22_H_24_O_11_ |
| 70 | Apigenin-7-O-glucuronide | C_21_H_18_O_11_ |
| 71 | 1,3,7-trihydroxy-6-methoxyxanthen-9-one | C_14_H_10_O_6_ |
| 72 | Cyanidin-3-hydroxybenzoyl-glucoside | C_28_H_25_O_13_+ |
| 73 | 1,2,3,6,7-Pentamethoxyxanthone | C_18_H_18_O_7_ |
| 74 | 1-Glycosyloxy-2-hydroxy-4-methoxyxanthone | C_20_H_20_O_10_ |
| 75 | Baicalin | C_21_H_18_O_11_ |
| 76 | 4-(beta-D-Glucopyranosyl)-7-methoxy-1,3,6-trihydroxy-9H-xanthene-9-one | C_20_H_20_O_11_ |
| 77 | 1,7-Dihydroxy-2,3-dimethoxyxanthone | C_15_H_12_O_6_ |
| 78 | 3-{[(2s,3r,4s,5s,6r)-4,5-dihydroxy-6-(hydroxymethyl)-3-{[(2s,3r,4r,5r,6s)-3,4,5-trihydroxy-6-methyloxan-2-yl]oxy}oxan-2-yl]oxy}-6,7-dihydroxy-2-methoxyxanthen-9-one | C_26_H_30_O_15_ |
| 79 | Ladanetin-6-O-β-D-glucoside | C_22_H_22_O_11_ |
| 80 | Gossypetin-3-O-rutinoside | C_27_H_30_O_17_ |
| 81 | Quercetin-7-O-rutinoside | C_27_H_30_O_16_ |
| 82 | Ternatumoside II | C_27_H_30_O_15_ |
| 83 | Nobiletin | C_21_H_22_O_8_ |
| 84 | Rutin | C_27_H_30_O_16_ |
| 85 | Delphinidin-3-O-(6''-O-p-coumaroyl)glucoside | C_30_H_27_O_14_+ |
| 86 | Isogentisin | C_14_H_10_O_5_ |
| 87 | Eriodictyol-5,3'-Di-O-glucoside | C_27_H_32_O_16_ |
| 88 | Eriodictyol-7-O-glucoside | C_21_H_22_O_11_ |
| 89 | Swertianolin | C_20_H_20_O_11_ |
| 90 | 6,8-Dihydroxy-1,2,3-trimethoxyxanthone | C_16_H_14_O_7_ |
| 91 | 1,4-dihydroxyxanthen-9-one | C_13_H_8_O_4_ |
| 92 | 2',4,4',6'-Tetrahydroxychalcone 4'-O-glucoside | C_21_H_22_O_10_ |
| 93 | Quercetin-3-O-(6''-O-p-Coumaroyl)galactoside | C_30_H_26_O_14_ |
| 94 | Nidulalin a | C_16_H_14_O_6_ |
| 95 | Eriodictyol-7-O-(6''-O-p-coumaroyl)glucoside | C_30_H_28_O_13_ |
| 96 | Montixanthone | C_14_H_10_O_6_ |
| 97 | 4-C-Glucose-1,3,6-trihydroxy-7-methoxyxanthone | C_20_H_20_O_11_ |
| 98 | Genistein 4'-O-Glucuronide | C_21_H_18_O_11_ |
| 99 | 1,3,6,7-Tetrahydroxyxanthone | C_13_H_8_O_6_ |
| 100 | Urolithin D | C_13_H_8_O_6_ |
| 101 | 6,7-Dimethoxy-4-chromanone | C_11_H_12_O_4_ |
| 102 | Isomucronulatol | C_17_H_18_O_5_ |
| 103 | Naringenin 7-Sulfate | C_15_H_12_O_8_S |
| 104 | Sibiricaxanthone A | C_24_H_26_O_14_ |
| 105 | 2-hydroxy-2-{10-hydroxy-5-methoxy-6-oxo-1h,2h-furo[2,3-c]xanthen-2-yl}propyl acetate | C_21_H_20_O_8_ |
| 106 | Mangiferin | C_19_H_18_O_11_ |
| 107 | Chrysin | C_15_H_10_O_4_ |
| 108 | Prunetin (5,4'-Dihydroxy-7-methoxyisoflavone) | C_16_H_12_O_5_ |
| 109 | Acacetin* | C_16_H_12_O_5_ |
| 110 | Tetahydroxyflavone-7-O-glucuronide | C_21_H_18_O_12_ |
| 111 | Luteolin 7-O-glucuronide | C_21_H_18_O_12_ |
| 112 | 2-Methoxy-4-hydroxy-6-(8Z-pentadecenyl)-benzene-1-O-acetate | C_24_H_38_O_4_ |
| 113 | Rubiadin | C_15_H_10_O_4_ |
| 114 | 1,2,5,7,8-pentahydroxy-3-methylanthracene-9,10-dione | C_15_H_10_O_7_ |
| 115 | 2,7-Dihydroxy-3,4,6-trimethoxyphenanthrene | C_16_H_16_O_4_ |
| 116 | α-Hydrojuglone glucoside | C_16_H_18_O_8_ |
| 117 | Embelin | C_17_H_26_O_4_ |
| 118 | Chrysophanol-9-anthrone | C_15_H_12_O_3_ |
| 119 | 1,4,8-Trihydroxynaphthalene-1-O-glucoside | C_16_H_18_O_8_ |
| 120 | isoemodin | C_15_H_10_O_5_ |
| 121 | Emodin | C_15_H_10_O_5_ |
| 122 | 7-Hydroxy-4-methyl-8-nitrocoumarin | C_10_H_7_NO_5_ |
| 123 | Avicennol | C_20_H_22_O_5_ |
| 124 | Lignans Machilin F | C_20_H_22_O_5_ |
| 125 | Decursinol | C_14_H_14_O_4_ |
| 126 | Aglacin K | C_24_H_30_O_9_ |
| 127 | Arctigenin | C_21_H_24_O_6_ |
| 128 | O-Feruloyl 4-hydroxycoumarin | C_19_H_14_O_6_ |
| 129 | 6-Methoxycoumarin | C_10_H_8_O_3_ |
| 130 | 8-Methoxy-6,7-Methylenedioxycoumarin | C_11_H_8_O_5_ |
| 131 | sanshodiol | C_20_H_22_O_6_ |
| 132 | (+)-Peusedanol | C_14_H_16_O_5_ |
| 133 | Isofraxidin | C_11_H_10_O_5_ |
| 134 | O-Feruloyl 7-hydroxycoumarin | C_19_H_14_O_6_ |
| 135 | Matairesinol | C_20_H_22_O_6_ |
| 136 | Daphnoretin Methyl Ether | C_20_H_14_O_7_ |
| 137 | 6,7-Dimethoxy-4-methylcoumarin | C_12_H_12_O_4_ |
| 138 | Coumarin | C_9_H_6_O_2_ |
| 139 | Herniarin | C_10_H_8_O_3_ |
| 140 | 7,8-Dihydroxy-4-phenylcoumarin | C_15_H_10_O_4_ |
| 141 | 1,4-Benzodioxin-6-propanol | C_11_H_12_O_3_ |
| 142 | Sesamin | C_20_H_18_O_6_ |
| 143 | Isoscopoletin-β-D-glucoside | C_16_H_18_O_9_ |
| 144 | Toddalolactone | C_16_H_20_O_6_ |
| 145 | 1',2'-epoxy-4-isobutyryl coniferol | C_14_H_18_O_5_ |
| 146 | 4-methoxy-5-hydroxymethylcoumarin | C_11_H_10_O_4_ |
| 147 | (-)-Pinoresinol glucoside | C_26_H_32_O_11_ |
| 148 | Sideretin (5,7,8-Trihydroxy-6-methoxycoumarin) | C_10_H_8_O_6_ |
| 149 | Polygalaxanthone XI | C_25_H_28_O_15_ |
| 150 | Polygalaxanthone V | C_26_H_30_O_15_ |
| 151 | Polygalaxanthone VII | C_27_H_32_O_16_ |
| 152 | 4-Nitrophenol | C_6_H_5_NO_3_ |
| 153 | Polygalaxanthone IX | C_25_H_28_O_14_ |
| 154 | 2-(2-Ethynyl-2,4-cyclopentadien-1-ylidene)ethenol | C_9_H_6_O |
| 155 | 3-Hydrocinnamyl-6-CinnamylGlucose | C_24_H_30_O_6_ |
| 156 | 3-O-Glucoside-3-hydroxy-y-butyrolactone | C_10_H_16_O_8_ |
| 157 | Aspidinol | C_12_H_16_O_4_ |
| 158 | Tetradecasphinganine | C_14_H_31_NO_2_ |
| 159 | Bellidifolin | C_14_H_10_O_6_ |
| 160 | Hexaethylene glycol | C_12_H_26_O_7_ |
| 161 | Senkyunolide M | C_16_H_22_O_4_ |
| 162 | gnetupendin A | C_22_H_20_O_5_ |
| 163 | 7-(4-Hydroxyphenyl)-1-phenyl-4-hepten-3-one | C_19_H_20_O_2_ |
| 164 | Senkyunolide C | C_12_H_12_O_3_ |
| 165 | 2,2-dimethylchromene-6-carboxylic acid* | C_12_H_12_O_3_ |
| 166 | 3,5-Dimethyl-2,3-dihydrobenzofuran | C_10_H_12_O |
| 167 | 3,5-di-tert-Butyl-4-hydroxyacetophenone | C_16_H_24_O_2_ |
| 168 | 2-benzoyl-4-[(2r)-1-hydroxy-7-methyl-3-methylideneoct-6-en-1-yl]benzene-1,3,5-triol | C_23_H_26_O_5_ |
| 169 | 2-benzoyl-4-[(2r)-2-hydroxy-7-methyl-3-methylideneoct-6-en-1-yl]benzene-1,3,5-triol | C_23_H_26_O_5_ |
| 170 | Dillapiol | C_12_H_14_O_4_ |
| 171 | Myricatomentogenin | C_20_H_22_O_5_ |
| 172 | Tributyl acetylcitrate | C_20_H_34_O_8_ |
| 173 | tetrahydrochromone M | C_20_H_22_O_5_ |
| 174 | Octadeca-2,9,12,15-tetraen-1-ol | C_18_H_30_O |
| 175 | tributyl phosphate | C_12_H_27_O_4_P |
| 176 | (diphenylphosphoroso)benzene | C_18_H_15_OP |
| 177 | 3,4-Methylenedioxycinnamaldehyde | C_10_H_8_O_3_ |
| 178 | 6-Beta-D-Glucopyranosyl-5,7-Dihydroxy-2-Isopropylchromone | C_18_H_22_O_9_ |
| 179 | 1,3,7-Trihydroxy-2-methoxyxanthone-glucose-arabinoside | C_25_H_28_O_15_ |
| 180 | 4-O-(2''-O-acetyl-6''-P-coumaroyl-β-D-glucopyranosyl)-P-coumaric acid | C_26_H_26_O_11_ |
| 181 | Dictyolactone | C_20_H_30_O_2_ |
| 182 | Rhizophorin C | C_20_H_30_O_2_ |
| 183 | 1,7-Dihydroxy-3,5,6-trimethoxyxanthone | C_16_H_14_O_7_ |
| 184 | 2-Pentadecanone | C_15_H_30_O |
| 185 | 6-Methoxy-2-(2-phenylethyl)chromone | C_18_H_16_O_3_ |
| 186 | (2-{[(2S)-2,3-dihydroxypropyl phosphonato]oxy}ethyl)trimethylazanium | C_8_H_20_NO_6_P |
| 187 | Polygalaxanthone B | C_18_H_18_O_7_ |
| 188 | Quinoline | C_9_H_7_N |
| 189 | 1,7-Dihydroxy-3,4-dimethoxyxanthone-glucoside | C_21_H_22_O_11_ |
| 190 | Indanone | C_9_H_8_O |
| 191 | 3,4-dihydroxy phenylethanol | C_8_H_10_O_3_ |
| 192 | Lanceolune B | C_12_H_12_O_4_ |
| 193 | 3-(3,4,5-trimethoxyphenyl)propanoic acid | C_12_H_16_O_5_ |
| 194 | 1,7-Dihydroxy-3,8-dimethoxyxanthone | C_15_H_12_O_6_ |
| 195 | 1,7-Dihydroxy-3,5,6-trimethoxyxanthone-glucoside | C_22_H_24_O_12_ |
| 196 | Antiarol | C_9_H_12_O_4_ |
| 197 | 1,3-Dihydroxy-2-methoxyxanthone-glucoside | C_20_H_20_O_10_ |
| 198 | 3,4,5-Trimethoxyphenol-1-O-β-D-glucoside | C_15_H_22_O_9_ |
| 199 | 3-Hydroxy-1,2-dimethoxyxanthone-glucoside | C_21_H_22_O_10_ |
| 200 | 1,3,7-Trihydroxy-2-methoxyxanthone-glucoside | C_20_H_20_O_11_ |
| 201 | Capillarisin | C_16_H_12_O_7_ |
| 202 | Isovanillin | C_8_H_8_O_3_ |
| 203 | 2',4'-Dihydroxyacetophenone | C_8_H_8_O_3_ |
| 204 | 1,2,3-Trimethoxy-7-hydroxyxanthone | C_16_H_14_O_6_ |
| 205 | Swerchirin | C_15_H_12_O_6_ |
| 206 | Maltol-glucoside | C_12_H_16_O_8_ |
| 207 | Dianthoside | C_12_H_16_O_8_ |
| 208 | Tetraethyleneglycol | C_8_H_18_O_5_ |
| 209 | 2,5-Dimethyl-7-hydroxychromone glucoside | C_17_H_20_O_8_ |
| 210 | 1,7-Dihydroxy-3,4-dimethoxyxanthone | C_15_H_12_O_6_ |
| 211 | epiloliolide | C_11_H_16_O_3_ |
| 212 | 4-hydroxyphenyl acrylaldehyde | C_9_H_8_O_3_ |
| 213 | 1,7-Dihydroxy-3,4-dimethoxyxanthone-glucose-rhamnoside | C_27_H_32_O_15_ |
| 214 | E-6,7-Dihydroxydihydroligustilide | C_12_H_16_O_4_ |
| 215 | 2-hydroxymethyl-D-ribono-γ-lactone | C_5_H_8_O_4_ |
| 216 | 1,7-bis(4-hydroxy-3-methoxyphenyl)hept-1-ene-3-ol | C_21_H_26_O_5_ |
| 217 | 2,6-Dimethoxy-4-hydroxyphenol-1-O-ß-D-glucopyranoside | C_14_H_20_O_9_ |
| 218 | 1,3-Dihydroxyxanthone-glucoside | C_19_H_18_O_9_ |
| 219 | Rubrofusarin-6-O-glucoside | C_21_H_22_O_10_ |
| 220 | 3,4-Dihydroxybenzaldehyde | C_7_H_6_O_3_ |
| 221 | 1,6,7-Trihydroxy-2,3-dimethoxyxanthone-glucoside | C_21_H_22_O_12_ |
| 222 | 3,6-Dihydroxy-1,2,7-trimethoxyxanthone | C_16_H_14_O_7_ |
| 223 | Butylphthalide | C_12_H_14_O_2_ |
| 224 | 1-(4-Hydroxy-3-methoxyphenyl)-7-phenylhept-4-en-3-one | C_20_H_22_O_3_ |
| 225 | Batatasin IV | C_15_H_16_O_3_ |
| 226 | 2,6-Di-tert-butylphenol | C_14_H_22_O |
| 227 | 1,3-Benzodioxole-5-nonanoic acid | C_16_H_22_O_4_ |
| 228 | Methyl (4E,8E,11E)-5,9,13-trimethyltetradeca-4,8,11-trienoate | C_18_H_30_O_2_ |
| 229 | (3,7-dimethyl-11-oxododeca-2,6-dienyl)acetate | C_16_H_26_O_3_ |
| 230 | 2,6-Di-tert-butyl-4-hydroxymethylphenol | C_15_H_24_O_2_ |
| 231 | (X)-1,2-Propanediol 1-O-B-D-Glucopyranoside | C_9_H_18_O_7_ |
| 232 | 2-Decanol | C_10_H_22_O |
| 233 | 2,3,5-trihydroxy-4-(phosphonooxy)hexanedioic acid | C_6_H_11_O_11_P |
| 234 | 1-Decanol | C_10_H_22_O |
| 235 | 4'-Methoxyresveratrol | C_15_H_14_O_3_ |
| 236 | ampelopsin C | C_42_H_32_O_9_ |
| 237 | (3E,5E,8Z,11Z)-10,13,15-trimethylheptadeca-3,5,8,11-tetraenoic acid | C_20_H_32_O_2_ |
| 238 | Ethyl (10Z,13Z)-hexadeca-10,13-dienoate | C_18_H_32_O_2_ |
| 239 | Moniliferanone A | C_24_H_34_O_4_ |
| 240 | (3E,5E,8Z,11Z)-7,10,15-trimethylheptadeca-3,5,8,11-tetraenoic acid | C_20_H_32_O_2_ |
| 241 | Glyceraldehyde | C_3_H_6_O_3_ |
| 242 | 2,6-Dimethoxybenzaldehyde | C_9_H_10_O_3_ |
| 243 | 4-(Beta-D-Glucopyranosyloxy)-2,4',6-Trihydroxybenzophenone | C_19_H_20_O_10_ |
| 244 | Machilusolide D | C_18_H_30_O_3_ |
| 245 | 9-hydroxy-8-{[(2S,3R,4S,5S,6R)-3,4,5-trihydroxy-6-(hydroxymethyl)oxan-2-yl]oxy}-1H,3H-naphtho[2,3-c]furan-1-one | C_18_H_18_O_9_ |
| 246 | p-Coumaric acid 4-O-sulfate | C_9_H_8_O_6_S |
| 247 | 1-(4-Methoxyphenyl)-1-propanol | C_10_H_14_O_2_ |
| 248 | 3,4-methylenedioxy cinnamyl alcohol | C_10_H_10_O_3_ |
| 249 | Terreic acid | C_7_H_6_O_4_ |
| 250 | Salicylaldehyde | C_7_H_6_O_2_ |
| 251 | 4-Hydroxyacetophenone | C_8_H_8_O_2_ |
| 252 | pholidotol A | C_16_H_16_O_5_ |
| 253 | Syringaldehyde; 4-Hydroxy-3,5-Dimethoxybenzaldehyde | C_9_H_10_O_4_ |
| 254 | 2-Acetylphloroglucinol | C_8_H_8_O_4_ |
| 255 | 6,8-dimethoxy-4a,9a-dihydro-9H-xanthen-9-one | C_15_H_14_O_4_ |
| 256 | 2,3,5,4'-Tetrahydroxystilbene-2-O-xyloside | C_19_H_20_O_8_ |
| 257 | 4,9-Dihydroxy-17-methoxy-2-oxatricyclo[13.2.2.13,7]icoSa-1(17),3,5,7(20),15,18-hexaen-10-one | C_20_H_22_O_5_ |
| 258 | 4-Hydroxybenzaldehyde | C_7_H_6_O_2_ |
| 259 | 4-Hydroxy-3-methoxy-benzaldehyde | C_8_H_8_O_3_ |
| 260 | Lumichrome | C_12_H_10_N_4_O_2_ |
| 261 | Octadecadienamide | C_18_H_33_NO |
| 262 | 1-Methylnicotinamide | C_7_H_9_N_2_O |
| 263 | N-Methylnicotinate | C_7_H_7_NO_2_ |
| 264 | N-(4-oxopentyl)-acetamide | C_7_H_13_NO_2_ |
| 265 | 1-Methylpiperidine-2-carboxylic acid | C_7_H_13_NO_2_ |
| 266 | 2-Amino-4,5-dihydro-1H-imidazole-4-acetic acid | C_5_H_9_N_3_O_2_ |
| 267 | 3-Chloroaniline | C_6_H_6_ClN |
| 268 | m-Aminophenylacetylene | C_8_H_7_N |
| 269 | 4-Methylazetidine-2-Carboxylic acid | C_5_H_9_NO_2_ |
| 270 | 2-Phenylacetamide | C_8_H_9_NO |
| 271 | N-benzylformamide | C_8_H_9_NO |
| 272 | Hexadecanamide | C_16_H_33_NO |
| 273 | Creatinine | C_4_H_7_N_3_O |
| 274 | 3-amino-2-naphthoic acid | C_11_H_9_NO_2_ |
| 275 | naphthisoxazol A | C_11_H_9_NO_2_ |
| 276 | 3,5-Dihydro-2H-Furo[3,2-C]Quinolin-4-One* | C_11_H_9_NO_2_ |
| 277 | 3-Indoleacrylic acid* | C_11_H_9_NO_2_ |
| 278 | N-Oleoylethanolamine | C_20_H_39_NO_2_ |
| 279 | Spermine | C_10_H_26_N_4_ |
| 280 | Zarzissine | C_5_H_5_N_5_ |
| 281 | Octadec-2-enamide | C_18_H_35_NO |
| 282 | 6-Deoxyfagomine | C_6_H_13_NO_2_ |
| 283 | alanine betaine | C_5_H_11_NO_2_ |
| 284 | Norephedrin 3-O-(2''-Phenylpropanyl)Glucoside | C_24_H_33_NO_6_ |
| 285 | 3-hydroxy-1-methylpyrrolidin-2-one | C_5_H_9_NO_2_ |
| 286 | 5-Hydroxy-2-pyrrolidinone | C_4_H_7_NO_2_ |
| 287 | 5-Hydroxypyridine-2(1H)-one | C_5_H_5_NO_2_ |
| 288 | Acetylcholine | C_7_H_16_NO_2_+ |
| 289 | N-Benzylmethylene isomethylamine | C_8_H_9_N |
| 290 | N-Cyclohexyl-1,3-benzothiazol-2-amine | C_13_H_16_N_2_S |
| 291 | L-Azetidine 2-carboxylic acid | C_4_H_7_NO_2_ |
| 292 | 3-Methylcytidine | C_10_H_15_N_3_O_5_ |
| 293 | Miserotoxin | C_9_H_17_NO_8_ |
| 294 | N-(3-hydroxy-4-methoxyphenethyl)-4-hydroxybutanamide | C_14_H_21_NO_4_ |
| 295 | 1-(Hydroxymethyl)hexahydro-1h-pyrrolizin-2-ol | C_8_H_15_NO_2_ |
| 296 | Pyrrolidin | C_4_H_9_N |
| 297 | L-Pipecolate | C_6_H_11_NO_2_ |
| 298 | L-Kynurenine | C_10_H_12_N_2_O_3_ |
| 299 | Choline | C_5_H_14_NO+ |
| 300 | Styrylamine | C_8_H_9_N |
| 301 | Piperidine | C_5_H_11_N |
| 302 | Valerine | C_8_H_15_NO_2_ |
| 303 | Stachydrine | C_7_H_13_NO_2_ |
| 304 | 4-(2-aminoethenyl)phenol | C_8_H_9_NO |
| 305 | N-(2-Hydroxy-4-methoxyphenyl)acetamide | C_9_H_11_NO_3_ |
| 306 | 4-Hydroxypipecolic acid | C_6_H_11_NO_3_ |
| 307 | Betaine | C_5_H_11_NO_2_ |
| 308 | 4-hydroxy-2-oxo-1,2-dihydroquinoline-3-carboxylic acid | C_10_H_7_NO_4_ |
| 309 | NSC614616 | C_6_H_10_N_2_O_2_ |
| 310 | Pseudotropine | C_8_H_15_NO |
| 311 | Tetradecyldiethanolamine | C_18_H_39_NO_2_ |
| 312 | Diethanolamine | C_4_H_11_NO_2_ |
| 313 | Cuscohygrine | C_13_H_24_N_2_O |
| 314 | 3,7,11-trimethyl-2-oxa-6,10,13-triazatricyclo[7.3.1.05,13]tridecane | C_12_H_23_N_3_O |
| 315 | 2-Methylserine | C_4_H_9_NO_3_ |
| 316 | 1-Aminopropan-2-ol | C_3_H_9_NO |
| 317 | Pipecolic acid | C_6_H_11_NO_2_ |
| 318 | Lycoperodine-1 | C_12_H_12_N_2_O_2_ |
| 319 | Indole-3-acetate | C_10_H_9_NO_2_ |
| 320 | L-Palmitoylcarnitine | C_23_H_45_NO_4_ |
| 321 | Α-hydroxyquinoline | C_9_H_7_NO |
| 322 | 8-hydroxyquinoline | C_9_H_7_NO |
| 323 | O-Acetyl-L-carnitine | C_9_H_17_NO_4_ |
| 324 | L-Carnitine | C_7_H_15_NO_3_ |
| 325 | 4,5,6-Trihydroxy-2-cyclohexen-1-ylideneacetonitrile | C_8_H_9_NO_3_ |
| 326 | N-((9E,11E)-13-methylpentadeca-9,11-dien-1-yl)acetamide | C_18_H_33_NO |
| 327 | Indoline | C_8_H_9_N |
| 328 | N-Acetylputrescine | C_6_H_14_N_2_O |
| 329 | Hordenine | C_10_H_15_NO |
| 330 | (+)-(1S,2S)-Pseudoephedrine | C_10_H_15_NO |
| 331 | 10-Formyltetrahydrofolate | C_20_H_23_N_7_O_7_ |
| 332 | 3-Indolepropionic acid | C_11_H_11_NO_2_ |
| 333 | 2-oxindole-3-acetic acid | C_10_H_9_NO_3_ |
| 334 | Decanoyl L-Carnitine | C_17_H_33_NO_4_ |
| 335 | 4-Hydroxy-3-methoxy-β-phenethylamine | C_9_H_13_NO_2_ |
| 336 | 5-Methoxyindoleacetate | C_11_H_11_NO_3_ |
| 337 | Dodecylcarnitine | C_19_H_37_NO_4_ |
| 338 | 2-(2-Benzothiazolylthio)ethanol | C_9_H_9_NOS_2_ |
| 339 | Hercynine | C_9_H_15_N_3_O_2_ |
| 340 | N-Methyl-2-(2-hydroxypropyl)-6-(2-hydroxybutyl)-Δ3-piperideine | C_13_H_25_NO_2_ |
| 341 | 2-Piperidone | C_5_H_9_NO |
| 342 | furan-2-carbohydrazide | C_5_H_6_N_2_O_2_ |
| 343 | 3-quinolinecarboxylic acid | C_10_H_7_NO_3_ |
| 344 | Echinopsine | C_10_H_9_NO |
| 345 | Isobutyryl carnitine | C_11_H_21_NO_4_ |
| 346 | N-Hydroxytryptamine | C_10_H_12_N_2_O |
| 347 | Cadaverine | C_5_H_14_N_2_ |
| 348 | 1-(2-(pyridin-3-yl)-2,3-dihydro-1H-pyrrol-1-yl)propan-1-one | C_12_H_14_N_2_O |
| 349 | Retronecine | C_8_H_13_NO_2_ |
| 350 | Indole-3-carboxaldehyde | C_9_H_7_NO |
| 351 | Dichotomine E | C_12_H_8_N_2_O_3_ |
| 352 | N-Feruloyltyramine 4'-glucoside | C_24_H_29_NO_9_ |
| 353 | Choline phosphate | C_5_H_15_NO_4_P+ |
| 354 | Agmatine | C_5_H_14_N_4_ |
| 355 | 5-Aminocycloheptane-1,2,3-triol | C_7_H_15_NO_3_ |
| 356 | N-(2-Hydroxyethyl)dodecanamide | C_14_H_29_NO_2_ |
| 357 | Methylguanidine | C_2_H_7_N_3_ |
| 358 | Butylamine | C_4_H_11_N |
| 359 | Synephrine; 4-[1-Hydroxy-2-(methylamino)ethyl]phenol | C_9_H_13_NO_2_ |
| 360 | o-Carboxy-5-hydroxytryptamine | C_11_H_12_N_2_O_3_ |
| 361 | N-Feruloylmethylagmatine | C_16_H_24_N_4_O_3_ |
| 362 | N1-Methyl-2-pyridone-5-carboxamide | C_7_H_8_N_2_O_2_ |
| 363 | N,N-cinnamoylbutanediamine | C_13_H_18_N_2_O |
| 364 | 10-Hydroxymethyllycaconitine | C_37_H_50_N_2_O_11_ |
| 365 | N,N'-Diferuloylputrescine | C_24_H_28_N_2_O_6_ |
| 366 | Acetryptine | C_12_H_14_N_2_O |
| 367 | Cephalanthrin A | C_17_H_12_N_2_O_4_ |
| 368 | Vincoside lactam | C_26_H_30_N_2_O_8_ |
| 369 | N-Acetylcadaverine | C_7_H_16_N_2_O |
| 370 | Valpromide | C_8_H_17_NO |
| 371 | 6-Hydroxynicotinate | C_6_H_5_NO_3_ |
| 372 | 3-(2'-Hydroxyl-phenyl)-4-(3H)-quinazolone | C_15_H_10_N_2_O_3_ |
| 373 | Hexylamine | C_6_H_15_N |
| 374 | Vanillylamine | C_8_H_11_NO_2_ |
| 375 | Cinnamamide | C_9_H_9_NO |
| 376 | isoindigotin | C_16_H_10_N_2_O_2_ |
| 377 | Indigo | C_16_H_10_N_2_O_2_ |
| 378 | 4-methoxybenzamide | C_8_H_9_NO_2_ |
| 379 | Crenatine | C_14_H_14_N_2_O |
| 380 | N1-acetylspermine | C_12_H_28_N_4_O |
| 381 | Canthin-6-one | C_14_H_8_N_2_O |
| 382 | Indole-3-carboxylic acid | C_9_H_7_NO_2_ |
| 383 | Indole-5-carboxylic acid | C_9_H_7_NO_2_ |
| 384 | Allantoin | C_4_H_6_N_4_O_3_ |
| 385 | Indolelactate | C_11_H_11_NO_3_ |
| 386 | Guanidinoacetate | C_3_H_7_N_3_O_2_ |
| 387 | N-Acetylisatin | C_10_H_7_NO_3_ |
| 388 | Indole-3-butyric acid | C_12_H_13_NO_2_ |
| 389 | Presenegenin | C_30_H_46_O_7_ |
| 390 | Tenuifolin | C_36_H_56_O_12_ |
| 391 | Fallaxsaponin A | C_35_H_54_O_11_ |
| 392 | Furanoeremophilane | C_15_H_22_O |
| 393 | Scutebarbolide C | C_22_H_32_O_8_ |
| 394 | 2,5,8-trimethyldeca-2,4,7-triene-1,10-diol | C_13_H_22_O_2_ |
| 395 | 16-oxo-21β-hydroxyserrat-14-en-3α-yl acetate | C_30_H_48_O_3_ |
| 396 | Dihydroisoalantolactone | C_15_H_22_O_2_ |
| 397 | 13-Hydroxygermacrone | C_15_H_22_O_2_ |
| 398 | 6α,10α-Dihydroxy-1-oxoeremophila-7(11),8(9)-dien-12,8-olide | C_15_H_18_O_5_ |
| 399 | Buddledin A | C_17_H_24_O_3_ |
| 400 | Mansonone N | C_16_H_22_O_4_ |
| 401 | 8,12-Epoxy-eremophila-9,11(13)-diene | C_15_H_22_O |
| 402 | Enantio-7(11)eudesmen-4-ol | C_15_H_26_O |
| 403 | Aristolone | C_15_H_22_O |
| 404 | Hinokiol | C_20_H_30_O_2_ |
| 405 | Liangshanin B | C_22_H_28_O_5_ |
| 406 | Damsinic acid | C_15_H_22_O_3_ |
| 407 | 6-O-Acetyl-3-O-(4-O-malonyl)-xylosylcycloastragenol | C_40_H_62_O_13_ |
| 408 | Eupalinolide B | C_24_H_30_O_9_ |
| 409 | 13-O-Feruloylplumieride | C_31_H_34_O_15_ |
| 410 | 5-hydroxy-4a-methyl-4,4a,5,6,7,8-hexahydronaphthalen-2(3h)-one | C_11_H_16_O_2_ |
| 411 | 5-hydroxy-1,4a-dimethyl-3,4,5,6,7,8-hexahydronaphthalen-2-one | C_12_H_18_O_2_ |
| 412 | Cycloartenol | C_30_H_50_O |
| 413 | Procurcumenol | C_15_H_22_O_2_ |
| 414 | serrat-14-ene-3,20,24,29-tetrol | C_28_H_46_O_4_ |
| 415 | Dendronobilin I | C_18_H_30_O_3_ |
| 416 | 8α-hydroxyeudesma-3,11(13)-dien-14-al | C_15_H_22_O_2_ |
| 417 | Polygodial | C_15_H_22_O_2_ |
| 418 | 3-Hydroxyurs-12-ene-27,28-dioic acid (Quinovic acid) | C_30_H_46_O_5_ |
| 419 | Dehydroilexhainanoside D | C_36_H_54_O_11_ |
| 420 | (-)-Menthone | C_10_H_18_O |
| 421 | Tagalsin Q | C_18_H_26_O_2_ |
| 422 | 2,3,23-Trihydroxyolean-12,18(19)-dien-28-oic acid-glucoside | C_36_H_56_O_10_ |
| 423 | Ganoderic Acid B8 | C_30_H_46_O_7_ |
| 424 | 3β,12-dihydroxy-13-methyl-podocarpane-8,10,13-tiene | C_18_H_26_O_2_ |
| 425 | Streterpene C | C_16_H_30_O_3_ |
| 426 | Verbenone | C_10_H_14_O |
| 427 | serratane-3α,14α,20β,21β,24,29-heptol | C_30_H_48_O_5_ |
| 428 | 11-hydroxy-3-oxo-4(5),6(7)-diene-eudesman-12-ol | C_15_H_22_O_2_ |
| 429 | JiangxiBaiyingsu I | C_18_H_28_O_3_ |
| 430 | Callicarpenal | C_16_H_26_O |
| 431 | phomophyllin H | C_15_H_22_O_2_ |
| 432 | 6,9-Dihydroxy-7-megastigmen-3-one | C_13_H_22_O_3_ |
| 433 | Irone | C_14_H_22_O |
| 434 | Cryptomeridiol | C_15_H_28_O_2_ |
| 435 | tripterifordin | C_20_H_30_O_3_ |
| 436 | 6-DeoxyCatalpol | C_15_H_22_O_9_ |
| 437 | Soyasapogenol B | C_30_H_50_O_3_ |
| 438 | Pisiferic acid | C_20_H_28_O_3_ |
| 439 | Glucosyl 7-methyl-3-methyleneoctane-1,2,6,7-tetraol | C_16_H_30_O_9_ |
| 440 | Lonchophylloid B | C_20_H_32_O_3_ |
| 441 | Ingenol | C_20_H_28_O_5_ |
| 442 | 2,6-Dimethyl-7-octene-2,3,6-triol | C_10_H_20_O_3_ |
| 443 | Dehydroabietic acid | C_20_H_28_O_2_ |
| 444 | Polygalasaponin Xxviii | C_53_H_84_O_24_ |
| 445 | (+)-Pimaric acid* | C_20_H_30_O_2_ |
| 446 | 3-Hydroxy-24-methylene-9,19-cyclolanostan-26-oic acid (Ambolic acid) | C_31_H_50_O_3_ |
| 447 | Isopimaric acid | C_20_H_30_O_2_ |
| 448 | Abietate* | C_20_H_30_O_2_ |
| 449 | Levopimaric acid | C_20_H_30_O_2_ |
| 450 | Bartsioside | C_15_H_22_O_8_ |
| 451 | Ent-15-oxo-2β,16,19-trihydroxypimar-8(14)-ene | C_20_H_32_O_4_ |
| 452 | 2-Formyl-3-hydroxy-A(1)-norlup-20(29)-en-28-oic acid (Colubrinic acid) | C_30_H_46_O_4_ |
| 453 | 8-Epiloganic acid | C_16_H_24_O_10_ |
| 454 | Estrane-3,17-diol | C_18_H_30_O_2_ |
| 455 | Gitoxigenin | C_23_H_34_O_5_ |
| 456 | 12(13→14)Abeospirost-5,13(18)-diene-1,3,15-triol (Dracaenogenin A) | C_27_H_40_O_5_ |
